# Data Driven Control of Vagus Nerve Stimulation for the Cardiovascular System: An *in Silico* Computational Study

**DOI:** 10.3389/fphys.2022.798157

**Published:** 2022-06-03

**Authors:** Andrew Branen, Yuyu Yao, Mayuresh V. Kothare, Babak Mahmoudi, Gautam Kumar

**Affiliations:** ^1^ Department of Chemical and Materials Engineering, San José State University, San José, CA, United States; ^2^ Department of Chemical and Biomolecular Engineering, Lehigh University, Bethlehem, PA, United States; ^3^ Department of Biomedical Informatics and Biomedical Engineering, School of Medicine, Emory University, Atlanta, GA, United States

**Keywords:** machine learning, closed-loop control, model predictive control, data-driven control, cardiac systems, vagus nerve stimulation therapy, long short-term memory, cardiovascular

## Abstract

Vagus nerve stimulation is an emerging therapy that seeks to offset pathological conditions by electrically stimulating the vagus nerve through cuff electrodes, where an electrical pulse is defined by several parameters such as pulse amplitude, pulse width, and pulse frequency. Currently, vagus nerve stimulation is under investigation for the treatment of heart failure, cardiac arrhythmia and hypertension. Through several clinical trials that sought to assess vagus nerve stimulation for the treatment of heart failure, stimulation parameters were determined heuristically and the results were inconclusive, which has led to the suggestion of using a closed-loop approach to optimize the stimulation parameters. A recent investigation has demonstrated highly specific control of cardiovascular physiology by selectively activating different fibers in the vagus nerve. When multiple locations and multiple stimulation parameters are considered for optimization, the design of closed-loop control becomes considerably more challenging. To address this challenge, we investigated a data-driven control scheme for both modeling and controlling the rat cardiovascular system. Using an existing *in silico* physiological model of a rat heart to generate synthetic input-output data, we trained a long short-term memory network (LSTM) to map the effect of stimulation on the heart rate and blood pressure. The trained LSTM was utilized in a model predictive control framework to optimize the vagus nerve stimulation parameters for set point tracking of the heart rate and the blood pressure in closed-loop simulations. Additionally, we altered the underlying *in silico* physiological model to consider intra-patient variability, and diseased dynamics from increased sympathetic tone in designing closed-loop VNS strategies. Throughout the different simulation scenarios, we leveraged the design of the controller to demonstrate alternative clinical objectives. Our results show that the controller can optimize stimulation parameters to achieve set-point tracking with nominal offset while remaining computationally efficient. Furthermore, we show a controller formulation that compensates for mismatch due to intra-patient variabilty, and diseased dynamics. This study demonstrates the first application and a proof-of-concept for using a purely data-driven approach for the optimization of vagus nerve stimulation parameters in closed-loop control of the cardiovascular system.

## 1 Introduction

Cardiovascular diseases are a prevalent health risk and financial burden. According to the annual statistics report from the American Heart Association (Benjamin et al., 2018), coronary heart disease (43.8%) is the leading cause of deaths attributable to cardiovascular disease (CVD) in the United States, followed by stroke (16.8%), high blood pressure (9.4%), heart failure (9.0%), diseases of the arteries (3.1%), and other CVDs (17.9%). It is projected that more than 130 million adults in the US population (45.1%) will have some form of CVD by 2035, leading to total costs of CVD reaching $1.1 trillion. The heart failure alone is projected to reach a total cost of $70 billion in 2030 (Heidenreich et al., 2013). Current pharmaceutical therapies lack adequate efficacy in treating cardiovascular diseases as demonstrated by the mortality rates (Ovbiagele et al., 2013; Savarese and Lund, 2017; Benjamin et al., 2018), which has motivated investigation efforts into alternative therapeutic approaches. Vagus nerve stimulation (VNS) has been identified and proposed as a potential therapy for a variety of cardiac conditions such as heart failure, atrial fibrillation, hypertension, and stroke (Capilupi et al., 2020). VNS involves sending electrical pulses through a cuff electrode to the vagus nerve, with the electrical pulse characterized by several parameters such as pulse width, pulse amplitude, and pulse frequency (Howland, 2014). A major challenge present in VNS delivery involves recruitment of specific fibers in the vagal nerve, as not all fibers have the same effect on the functioning of the cardiac system. A recent study has demonstrated the ability to recruit specific fiber types through the use of different stimulation locations in a rat heart (Plachta et al., 2014).

Another challenge present in delivering VNS therapy involves the optimal selection of VNS parameters to achieve a desired physiological response. Currently, VNS parameters are determined through manual titration in an open-loop configuration, as was used in the three clinical trials that investigated VNS for the treatment of heart failure (Premchand et al., 2014; Zannad et al., 2015; Gold et al., 2016). These clinical trials reached different conclusions regarding the efficacy of VNS. The different conclusions could be attributed to the different operating regimes for each trial, leading to the suggestion of finding optimal VNS parameters in future trials to clearly evaluate the efficacy of VNS (Asad and Stavrakis, 2019).

To address the challenge of finding the optimal VNS parameter selection, studies investigating the closed loop control of VNS stimulation has been accomplished in previous studies performed in sheep by using a proportional-integral controller design (Ugalde et al., 2015), and state-space transition models (Romero-Ugalde et al., 2017). Other studies have investigated using proportional-integral controller designs to control the heart rate of rats (Greenwald et al., 2016), pigs (Tosato et al., 2006), and dogs (Zhang et al., 2002). The previously discussed controllers only controlled the heart rate, and did not control multiple physiological outputs such as heart rate and blood pressure. Further, these controller studies did not optimize multiple input stimulation parameters, nor did they consider the possibility of different stimulation location sites. A recent *in silico* study developed a cardiac model of a rat heart with the influence of VNS and used a model predictive control (MPC) framework to optimize multiple VNS parameters (pulse width and pulse frequency) at multiple locations to control the heart rate and blood pressure simultaneously (Yao and Kothare, 2020).

For this application of MPC, there are challenges revolving around the development and validation of *in silico* cardiac models. Often such an approach becomes a challenging task in selecting the correct dynamical equations that govern the cardiac system, and then fitting the parameters to those specific equations. Such tasks can be guided by a deep mechanistic understanding of the system, however the definition of the system can vary, as shown by the variety of *in silico* cardiac models in the literature that range from modeling the individual neuronal cells in a cardiac tissue (Mangoni et al., 2006), to modeling the whole cardiovascular system as a pump (Suga et al., 1973). There have been some models that incorporate the effects of extrinsic stimulation on the cardiac system, such as simulating an orthostatic response in a human cardiovascular system (Melchior et al., 1992). However, most *in silico* computational models do not include the necessary variables to account for physiological changes mediated through VNS, leaving a challenging task for their application in VNS parameter optimization. Further, such models may be difficult to validate in experiments due to the variability of fiber recruitment in the vagus nerve. Adding to the challenge of using full-scale *in silico* physiological models is the computational expense associated with simulating these models for real-time closed-loop control.

Data driven modeling techniques are a viable approach that address the challenges associated with the previously described *in silico* physiological models. A common approach for data driven modeling includes machine learning, which can learn a compact representation of the nonlinear dynamics present in a variety of systems (Goodfellow et al., 2016). There are no underlying assumptions about the data fed to train the network or the distribution of the data fed to the network. Together, these features have led to the widespread application of machine learning for the modeling of nonlinear dynamical systems. More specifically, recurrent neural networks (RNNs) are well suited for time-series modeling shown by their state-of-the-art performance in challenging applications that include forecasting river flows (Sahoo et al., 2019), power usage in residential areas (Kong et al., 2017), and short-term traffic patterns (Zhao et al., 2017). Of noteworthy importance in these applications, a RNN was consistently shown to give better predictive performance of the time-series data when compared to a simple feedforward neural network. Thus, the hidden state embodied in a RNN improves the learning of intrinsic temporal symmetries that allow for more accurate predictions of future time-series data. Previously, long short-term memory (LSTM) neural networks which are a type of RNN, have been used to model the effect of current injection on a pyramidal neuron (Plaster and Kumar, 2019). Additionally LSTMs have been shown to be less computationally expensive for function evaluation, which motivates their application in real-time control of the cardiovascular system.

In this paper, we demonstrate a computationally efficient data-driven closed-loop control scheme to control the heart rate (HR) and the mean arterial blood pressure (MAP) in an *in silico* physiological model of a rat heart (Yao and Kothare, 2020). The approach presented in this paper is a “mimic” of how models and controllers could be developed from real data. In the absence of real-data, we create “simulated” or “artificial” data sets from a biophysical model and demonstrate the entire approach of model and controller development on this simulated/synthetic data. Once validated on this simulated system, the approach has a higher confidence of being relevant when applied to real data from a clinical or animal experimental setting. Specifically, we develop and use a LSTM model in a MPC framework to optimize the VNS parameters to achieve the desired set points in HR and MAP. We formulate unique optimization problems that consider different physiological contexts, and discuss their influence on the closed-loop controller performance. Our results show that our designed controllers lead to set point tracking with nominal offset for both HR and MAP. Next, we modify the *in silico* physiological model to exhibit intra-patient variability, caused by a unique concentration of neuronal fiber recruitment at the cuff electrode. To account for the intra-patient variability in the closed-loop control formulation, we include additional constraints in the optimization problem, thus enabling the control of the physiological variables without training another LSTM on the data from the modified *in silico* physiological model. We investigate this approach further by modifying the controlled *in silico* physiological model to demonstrate a case of an elevated sympathetic nervous system and a decreased vagal tone, similar to a diseased state. Through simulations, we show that our closed-loop control design can efficiently control HR and MAP for both the intra-patient and diseased state systems while using the same previously trained LSTM in designing the controller. Together, we demonstrate a novel computationally efficient data-driven closed-loop VNS design for modeling and controlling HR and MAP, which could potentially be translated to animal experiments for real-time control of the cardiac system.

## 2 Results

### 2.1 Data-Driven Mapping of VNS Parameters to the Heart Rate and Mean Arterial Blood Pressure

We recently developed a novel data-driven machine learning-based computational modeling approach to map the effect of VNS on the heart rate (HR) and the mean arterial blood pressure (MAP) (Branen et al., 2021). Briefly, we used a published computational model of the rat cardiovascular system (Yao and Kothare, 2020) to generate synthetic data by varying three VNS locations, pulse width, and stimulation frequency and measuring the effect of VNS parameters on HR and MAP (see the details of the *in silico* physiological model and the range of the VNS parameters in Materials and Methods Section 4.1). We then trained several neural networks, including recurrent neural networks (RNNs) and long short-term memory (LSTM) network, on this synthetic data by systematically varying the model hyperparameters, such as the number of hidden layers, hidden-state dimensionality, and activation functions, and compared the performance of the models in predicting HR and MAP in response to VNS. Our results showed that a single hidden layer LSTM network with a hidden-state dimensionality of 10 and a hyperbolic tangent activation function led to the best performance (Branen et al., 2021). Although we published these modeling results in Branen et al. (2021), for the completeness of this manuscript, we provide the details of our modeling approach and the comparison of various trained neural networks in the Materials and Methods Section 4.4.

**FIGURE 1:**
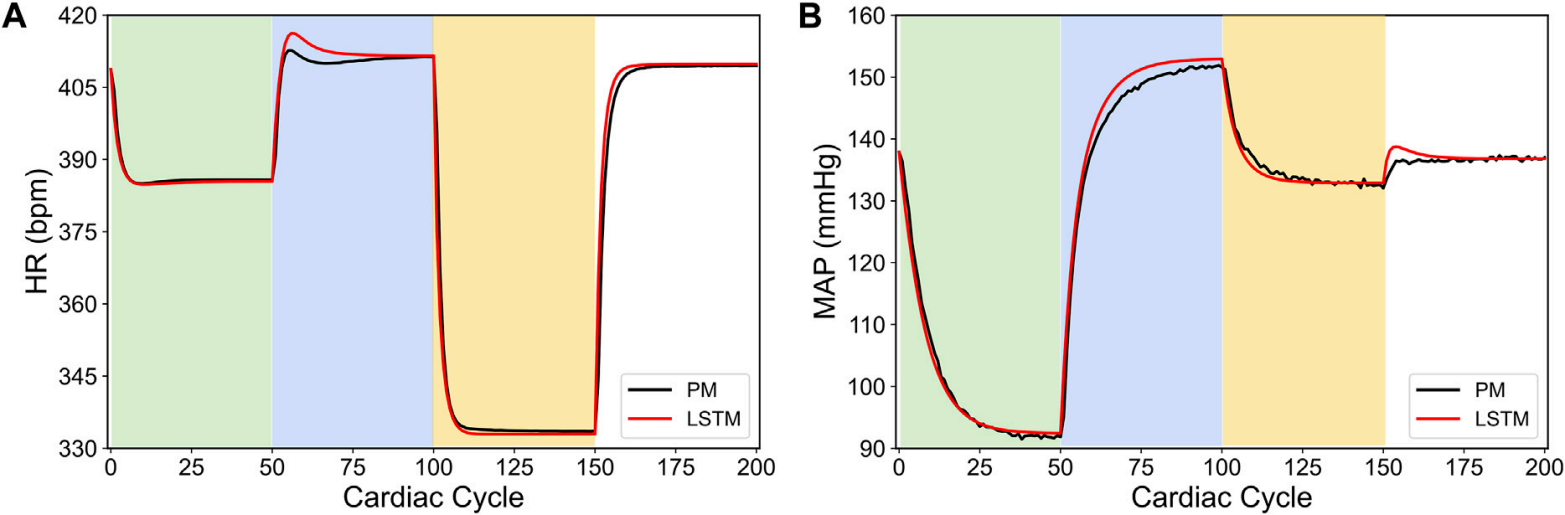
LSTM predictions (shown in red) compared to the output from the *in silico* physiological model of the rat cardiac system (PM, shown in black) for 200 simulated cardiac cycles of **(A)** heart rate and **(B)** mean arterial blood pressure (MAP). For cycles 1–50 (light green), location 1 was active with a pulse width of 0.36 ms and pulse frequency of 31 Hz. For cycles 51–100 (light blue), location 2 was active with a pulse width 0.19 ms and a pulse frequency 17 Hz. For cycles 101–150 (yellow), location 3 was active with a pulse width of 0.42 ms and a pulse frequency of 47 Hz. The last 50 cycles did not have any locations active.

Figure 1 shows a specific prediction of HR and MAP by our LSTM model and its comparison with the *in silico* physiological model (ground truth data), where we have varied the VNS parameters after every 50 cardiac cycles. As shown in this figure, the LSTM model is able to predict the response of the *in silico* physiological model with high accuracy, following the selection of time-varying VNS parameters. Notably, this predictive task requires the trained LSTM to recursively predict all 200 cardiac cycles, given the initial HR and MAP and the VNS parameters for all 200 cardiac cycles. In this way, the LSTM has correctly learned the mapping of the VNS parameters to the physiological effect on HR and MAP. Importantly, our LSTM model took 2.10 s to predict HR and MAP for 100 cardiac cycles on a local PC machine (Intel(R) Core I7-9700 CPU 3.00 GHz with 16 GB RAM) compared to 19.99 s by the *in silico* physiological model.

In conclusion, our results in Branen et al. (2021) demonstrated a computationally efficient data-driven machine learning approach to predict HR and MAP in response to VNS stimulation with high accuracy directly from the data. In the remainder of this paper, we will use our LSTM model to demonstrate how this model can be used to design a model-based optimal control strategy to regulate HR and MAP by optimizing the VNS parameters in a feedback-based closed-loop framework.

### 2.2 Model-Based Optimal Control Framework to Optimize VNS Parameters for Cardiac System

As described in the previous section, the *in silico* physiological model of a rat cardiovascular system (Yao and Kothare, 2020) has three distinct VNS locations, with each location having two design parameters, i.e., the pulse width and the stimulation frequency. These different stimulation locations, along with the pulse width and stimulation frequency as the design parameters at each location, have a distinctive effect on cardiac physiology, such as heart rate (HR) and mean arterial blood pressure (MAP). The presence of several stimulation design parameters raises the challenge of optimizing these parameters to achieve a desired physiological response.

**FIGURE 2:**
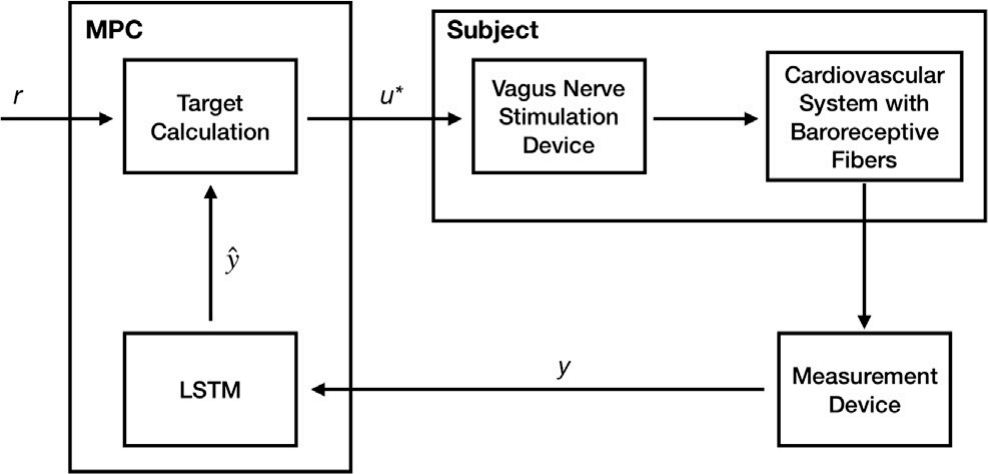
A Schematic showing our model-based predictive closed-loop optimal control framework for optimizing the vagus nerve stimulation (VNS) parameters to control multiple cardiac physiological biomarkers such as heart rate (HR) and mean arterial blood pressure (MAP). Here, a long short-term memory (LSTM) based recurrent neural network model has been used to make predictions of HR and MAP in response to VNS and optimize the VNS parameters within the model predictive control (MPC) framework.

In an experimental/clinical setting, the choice of these stimulation design parameters is typically accomplished manually based on the clinical experience. In order to automate this process, we developed a closed-loop optimal control approach to optimize the VNS parameters to achieve the desired HR and MAP in an *in silico* physiological model of the rat cardiac system using the model predictive control (MPC) framework. Figure 2 shows a schematic of our closed-loop control framework with the model-based controller utilizing predictions from the trained LSTM.

Briefly, MPC is an optimal control strategy that uses a model of the system in designing and optimizing the control actions. Specifically, at the current time, the measurements of the outputs are obtained from the system, and a model of the system is used to predict the system’s outputs in the future over a specified time horizon (*prediction horizon*) in terms of the current and future control actions over a specified time horizon (*control horizon*). Then an optimization problem is formulated and solved to minimize the difference between the predicted model outputs and the desired outputs over the prediction horizon by optimizing the VNS parameters over the control horizon. The first computed optimal control action is implemented on the system, and the prediction and control horizons are receded by one time-step in the future. This process is repeated until the system is driven to the desired outputs. One of the significant advantages of using MPC over other optimal control approaches in controlling the cardiac system is its flexibility in incorporating physiological constraints and uncertainties explicitly in the optimization problem formulation.

To demonstrate our approach, we considered the *in silico* physiological model of the rat cardiovascular system (Yao and Kothare, 2020) as the physiological cardiac system to be controlled. We adapted the MPC framework to design and optimize the VNS parameters—pulse width, stimulation frequency, and three VNS locations (i.e., 6 input parameters)—to control HR and MAP in the *in silico* physiological model of the rat cardiac system. To design our model-based controller, we used the LSTM model presented in the previous section as a predictive model of the *in silico* physiological model of the rat cardiovascular system and formulated the following optimization problem to be solved at each cardiac cycle:

$$\begin{array}{cccc} & \min & & J\left(k\right)\\ & \vec{u}\left(k+j-1|k\right),\;j=1,2,\ldots ,N_{c}\\ & s.t. & & {\vec{u}}_{min}\leq \vec{u}\left(k+j-1|k\right)\leq {\vec{u}}_{max},j\in \left[1,N_{c}\right]\\ & & & \hat{\vec{y}}\left(k+i|k\right)=f_{NN}\left(\hat{\vec{y}}\left(k+i-1|k\right),\vec{u}\left(k+i-1|k\right)\right),i\in \left[1,N_{p}\right]\\ & & & \hat{\vec{y}}\left(k|k\right)={\vec{y}}_{0}\left(k\right) \end{array}$$ (1)

Here, *f*_*NN*_(⋅, ⋅) denotes the LSTM model described in the previous section, $\hat{\vec{y}}\left(k+i|k\right)$ is the vector of predicted physiological outputs (i.e., HR and MAP) from the LSTM model at the discrete time (i.e., cardiac cycle) *k* + *i*, given that the outputs are measured from the *in silico* physiological model at the current time *k*, *N*_*c*_ and *N*_*p*_ are the control horizon and prediction horizon, respectively, and $\vec{u}\left(k+i|k\right)$ denotes the vector of VNS input parameters (i.e., the pulse width and the stimulation frequency at three VNS locations) at a future time *k* + *i*, given that the outputs are measured from the *in silico* physiological model at the current time *k*. ${\vec{y}}_{0}\left(k\right)$ is the vector of measured HR and MAP from the *in silico* physiological model at the current time (or cardiac cycle) *k* and J(k) is the user defined scalar cost function. In the optimization problem (1), the first constraint represents the upper and lower bounds on the VNS parameters over the control horizon. The second constraint states that the evolution of outputs over the prediction horizon must satisfy the LSTM model dynamics. The last constraint states that the current outputs of LSTM model are same as the measured outputs from the *in silico* physiological model at time *k*.

#### 2.2.1 Sparsity Promoted Closed-Loop VNS Design

As noted in the description of the optimization problem (1), the manipulated (or designed) VNS inputs consist of three VNS locations, with each location having two VNS design parameters, the pulse width, and the stimulation frequency. The presence of discrete or integer variables (locations of VNS) requires the formulation of the optimization problem (1) in the form of a well-known mixed-integer programming problem, which is typically computationally expensive to solve (Trespalacios and Grossmann, 2014). To keep our control strategy computationally efficient while achieving a similar performance compared to the mixed-integer programming solution, we formulated the cost function in the optimization problem (1) by introducing a *L*_1_ penalty on the VNS parameters as follows:

$$J\left(k\right)=\overset{N_{p}}{\underset{i=1}{\sum }}{\left(\vec{r}\left(k+i\right)-\hat{\vec{y}}\left(k+i|k\right)\right)}^{T}Q\left(\vec{r}\left(k+i\right)-\hat{\vec{y}}\left(k+i|k\right)\right)+\lambda \overset{N_{c}}{\underset{j=1}{\sum }}\|\vec{u}\left(k+j-1|k\right){\|}_{1}.$$ (2)

Here, the first term in the sum is a weighted quadratic cost defining the error between the target output (or reference) to be achieved by the controller and the predicted output from the LSTM model over the prediction horizon *N*_*p*_. $\vec{r}\left(k+i\right)\in R^{2\times 1}$ is a vector of the time varying target output (HR and MAP) at discrete time *k* + *i* and $Q\in R^{2\times 2}$ is the weighting matrix emphasizing the importance of specific outputs. The second term in the sum is a *L*_1_ cost on the manipulated inputs (the pulse width and stimulation frequency at three VNS locations) over the control horizon *N*_*c*_. The parameter *λ* defines the importance of these two terms in the cost function. We define the *L*_1_ cost as

$$\overset{N_{c}}{\underset{j=1}{\sum }}\|\vec{u}\left(k+j-1|k\right){\|}_{1}=\overset{N_{c}}{\underset{j=1}{\sum }}\overset{6}{\underset{i=1}{\sum }}|u_{i}\left(k+j-1|k\right)|.$$ (3)

Here, |⋅| represents the absolute value of the argument. *L*_1_ penalty in the cost function is well-known to introduce sparsity in the optimization variables, both in the control community and machine-learning community (Vidaurre et al., 2013).

To solve the optimization problem (1) with the cost function (2), we set *N*_*p*_ = 10, *N*_*c*_ = 5, and *λ* = 0.001. The initial measured HR and MAP from the physiological rat cardiac model were 409 (bpm) and 138 (mmHg), respectively. We selected the heart rate going from 392 (bpm) to 346 (bpm) followed by 393 (bpm), and mean arterial blood pressure going from 111 (mmHg) to 144 (mmHg) followed by 125 (bpm) with each pair lasting for 50 cardiac cycles as the desired set point trajectory to be followed by the controller.

**FIGURE 3:**
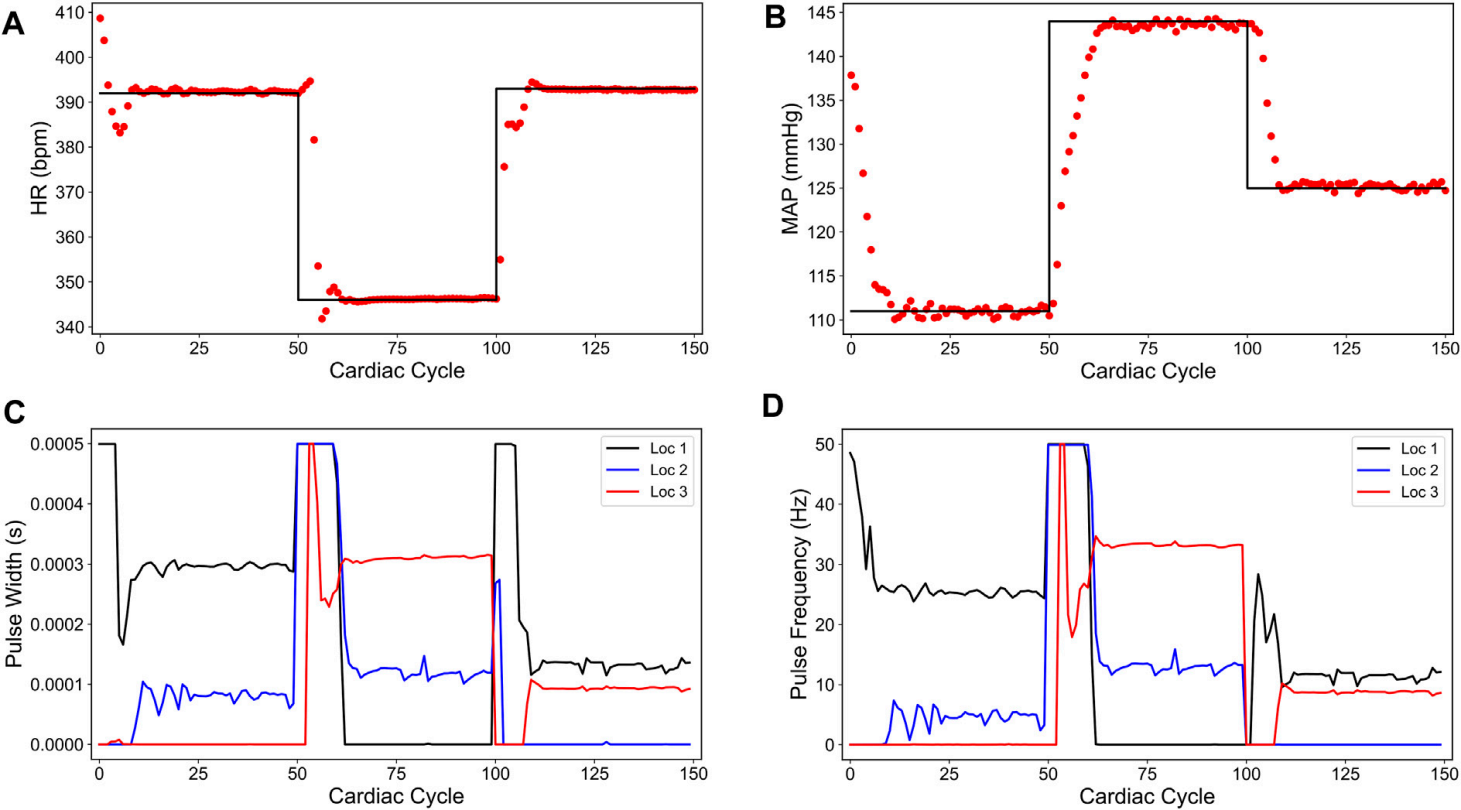
Sparsity-promoted closed-loop control of the heart rate (HR) and the mean arterial blood pressure (MAP) of the *in silico* physiological model of the rat cardiac system. **(A)** and **(B)** show the closed-loop control response from the *in silico* physiological model (shown in red dots) and the target (or reference) for the controller (shown in black line) for HR and MAP, respectively. **(C)** and **(D)** show the pulse width and the stimulation frequency, respectively, designed by the controller at each VNS location.

Figure 3 shows our simulation results on the sparsity promoted closed-loop VNS design to control HR and MAP simultaneously. In Figures 3A,B, we note that the designed controller efficiently drove the *in silico* physiological model to the desired time-varying set points with minimal to no steady-state offset for HR and MAP, respectively. Figures 3C,D show the optimized pulse width and stimulation frequency, respectively, delivered to the *in silico* physiological model at each cardiac cycle. As noted in Figures 3C,D, the controller activated locations 1 and 2 in the first and the last 50 cardiac cycles and locations 2 and 3 in the second 50 cardiac cycles to achieve the desired set points in each 50 cycles period.

We also note in Figures 3C,D that the controller actions are oscillating slightly. A possible reason for this behavior may be due to influence from the MAP variable, which exhibits fluctuations even at the steady-state (see Figure 1). Since there is no cost term forcing a constant solution on the controller inputs, the controller attempts to compensate for this fluctuation by altering its actions, thus leading to similar fluctuations in the controller actions. Additionally, it is worth noting that the LSTM had a higher error for MAP predictions (compared to HR) for this reason.

In summary, we showed a data-driven model-based closed-loop control strategy to optimize the VNS parameters with multiple stimulation location sites, leading to a sparse selection of VNS parameters in controlling cardiac physiology efficiently. This strategy may find applications in developing efficient closed-loop VNS therapy for cardiac diseases by minimizing the side effects of specific stimulation locations. Although we have not considered all possible physiological constraints in formulating our optimization problem, the framework is general enough to introduce additional physiological constraints within the optimization problem.

#### 2.2.2 Minimum Energy-Based Closed-Loop VNS Design

In the previous section, we presented a sparsity-promoted closed-loop VNS strategy that selectively stimulated specific VNS locations to control the heart rate (HR) and mean arterial blood pressure (MAP). Although this strategy could potentially minimize the location-specific side effects induced by VNS, the bang-bang nature of this strategy (i.e., delivering VNS at the maximum allowable pulse width and frequency) may damage tissues over a longer period of VNS application (Agnew et al., 1989). To minimize the duration of the applied VNS at the maximum allowable pulse width and frequency, we designed a closed-loop optimal control strategy by minimizing the stimulation energy required to drive HR and MAP to the desired set points. To implement this strategy, we chose the following cost function in the optimization problem (1):

$$J\left(k\right)=\overset{N_{p}}{\underset{i=1}{\sum }}{\left(\vec{r}\left(k+i\right)-\hat{\vec{y}}\left(k+i|k\right)\right)}^{T}Q\left(\vec{r}\left(k+i\right)-\hat{\vec{y}}\left(k+i|k\right)\right)+\lambda \overset{N_{c}}{\underset{j=1}{\sum }}\|\vec{u}\left(k+j-1|k\right){\|}_{2}^{2}.$$ (4)

Here, the first term in the sum is a weighted quadratic cost defining the error between the target output (or reference) to be achieved by the controller and the predicted output from the LSTM model over the prediction horizon *N*_*p*_. $\vec{r}\left(k+i\right)\in R^{2\times 1}$ is a vector of the time varying target output (HR and MAP) at discrete time *k* + *i* and $Q\in R^{2\times 2}$ is the weighting matrix emphasizing the importance of specific outputs. The second summation term is a *L*_2_ cost on the manipulated inputs (the pulse width and stimulation frequency at three VNS locations) over the control horizon *N*_*c*_. The parameter *λ* defines the importance of these two terms in the cost function. It should be noted that the cost function (4) is same as the sparsity promoted cost function (2) described in the previous section except we replaced the *L*_1_ penalty in (2) by a *L*_2_ penalty. We define the *L*_2_ cost as

$$\overset{N_{c}}{\underset{j=1}{\sum }}\|\vec{u}\left(k+j-1|k\right){\|}_{2}^{2}=\overset{N_{c}}{\underset{j=1}{\sum }}{\vec{u}}^{T}\left(k+j-1|k\right)R\vec{u}\left(k+j-1|k\right).$$ (5)

Here (⋅)^*T*^ represents a vector transpose and $R\in R^{6\times 6}$ is the weighting matrix defining the importance of individual inputs. *L*_2_ penalty in the cost function is well-known to achieve a minimum energy solution by suppressing large amplitude control actions (Kwakernaak et al., 1974).

To solve the optimization problem (1) with the cost function (4), we set *N*_*p*_ = 10, *N*_*c*_ = 5, and *λ* = 0.001. We set the design parameters *Q* and *R* to an identity matrix of appropriate dimensions. The initial measured HR and MAP from the physiological rat cardiac model were 409 (bpm) and 138 (mmHg), respectively. We selected the heart rate going from 392 (bpm) to 346 (bpm) followed by 393 (bpm), and mean arterial blood pressure going from 111 (mmHg) to 144 (mmHg) followed by 125 (mmHg) with each variable lasting for 50 cardiac cycles as the desired set point trajectory to be followed by the controller.

**FIGURE 4:**
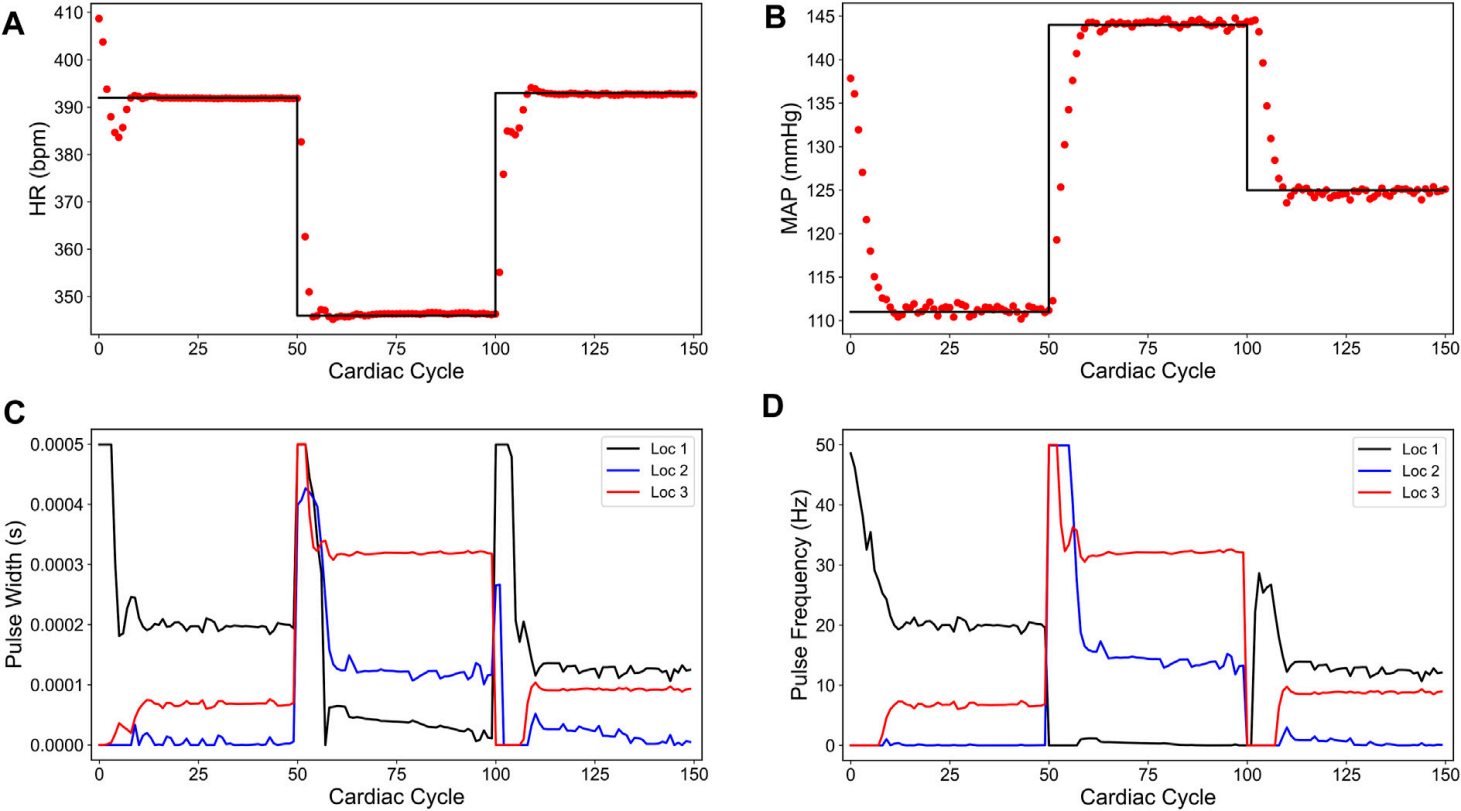
Minimum energy-based closed-loop control of the heart rate (HR) and the mean arterial blood pressure (MAP) of the *in silico* physiological model of the rat cardiac system. **(A)** and **(B)** show the closed-loop control response from the *in silico* physiological model (shown in red dots) and the target (or reference) for the controller (shown in black line) for HR and MAP, respectively. **(C)** and **(D)** show the pulse width and the stimulation frequency, respectively, designed by the controller at each VNS location.

Figure 4 shows our simulation results on the minimum energy-based closed-loop VNS design in controlling HR and MAP of the *in silico* physiological model of rat cardiovascular system simultaneously. In Figures 4A,B, we show that the designed controller can efficiently drive the *in silico* physiological model to the desired time-varying set points with minimal to no offset for HR and MAP, respectively. We note that the output performance of the designed controller is similar to the sparsity promoted design described in the previous section, with slight oscillations in MAP. Figures 4C,D show the optimized pulse width and stimulation frequency, respectively, delivered to the physiological model at each cardiac cycle. As noted in Figures 4C,D, the controller activated all the locations to achieve the desired set points in each 50 cycle period as opposed to the sparsity-promoted design shown in Figures 3C,D. More importantly, the designed controller selected minimum values on the VNS parameters (pulse width and frequency) required to drive HR and MAP to the desired set points by minimizing the input energy.

In summary, we showed a data-driven model-based closed-loop control strategy to optimize the VNS parameters with multiple stimulation location sites, leading to a minimum energy based selection of VNS parameters in controlling cardiac physiology efficiently. This strategy may find applications in developing efficient closed-loop VNS therapy for cardiac diseases by enhancing the battery life of the VNS device. Moreover, this strategy could easily be combined with the sparsity promoted closed-loop VNS design by including an additional *L*_1_ penalty term in the cost function (4).

#### 2.2.3 Minimum Overshoot Based Closed-Loop VNS Design

In the previous two closed-loop VNS designs, we noted that the controller actions oscillate around the steady-state values (see Figures 3C,D, 4C,D). To eliminate or reduce the oscillatory behavior in the controller actions, we included an additional term in the cost function that penalizes the deviation in inputs, when comparing the current and future stimulation parameters to the previously implemented optimal stimulation parameters to the *in silico* physiological model. Particularly, we formulated the cost function J(k) in the optimization problem (1) as follows:

$$\begin{array}{ll} J\left(k\right) & =\overset{N_{p}}{\underset{i=1}{\sum }}{\left(\vec{r}\left(k+i\right)-\hat{\vec{y}}\left(k+i|k\right)\right)}^{T}Q\left(\vec{r}\left(k+i\right)-\hat{\vec{y}}\left(k+i|k\right)\right)\\ & \;+\lambda_{1}\overset{N_{c}}{\underset{j=1}{\sum }}\|\vec{u}\left(k+j-1|k\right){\|}_{1}+\lambda_{2}\overset{N_{c}}{\underset{j=1}{\sum }}\|\vec{u}\left(k+j-1|k\right)-{\vec{u}}_{k-1}{\|}_{1}. \end{array}$$ (6)

Here, the first two terms of the cost function (6) are same as the sparsity promoted cost function (2), as described in Section 2.2.1. The last term in the cost function (6) is the difference between the current and future control inputs over the control horizon *N*_*c*_ and the last applied optimal control input ${\vec{u}}_{k-1}$ on the *in silico* physiological model of the rat cardiovascular system (i.e., the physiological system). *λ*_1_ and *λ*_2_ are the weighting parameters.

By including the difference in controller action term in the cost function (6), we forced the controller actions to take values close to the last implemented optimal control action on the *in silico* physiological model. As a result of the constant controller actions, the physiological variables should exhibit minimal overshoot as they gradually reach the setpoint. Additionally, any fluctuations in HR or MAP at the setpoint are the result of intrinsic model dynamics. The inclusion of the L1 cost term in the cost function (6) serves to maintain a sparse solution.

For the closed-loop simulation, we set *N*_*c*_ = 5, *N*_*p*_ = 10, *λ*_1_ = 0.001, and *λ*_2_ = 0.00005. It should be noted that different selections of *N*_*c*_ and *N*_*p*_ can lead to similar performance, provided that the weights (*λ*_1_, *λ*_2_) are appropriately selected. The initial values of HR and MAP were 409 (bpm) and 138 (mmHg), respectively. The values for *Q* and *R* were again an identity matrix of the appropriate dimensions. The duration and set points for both HR and MAP were the same as mentioned in previous sections.

**FIGURE 5:**
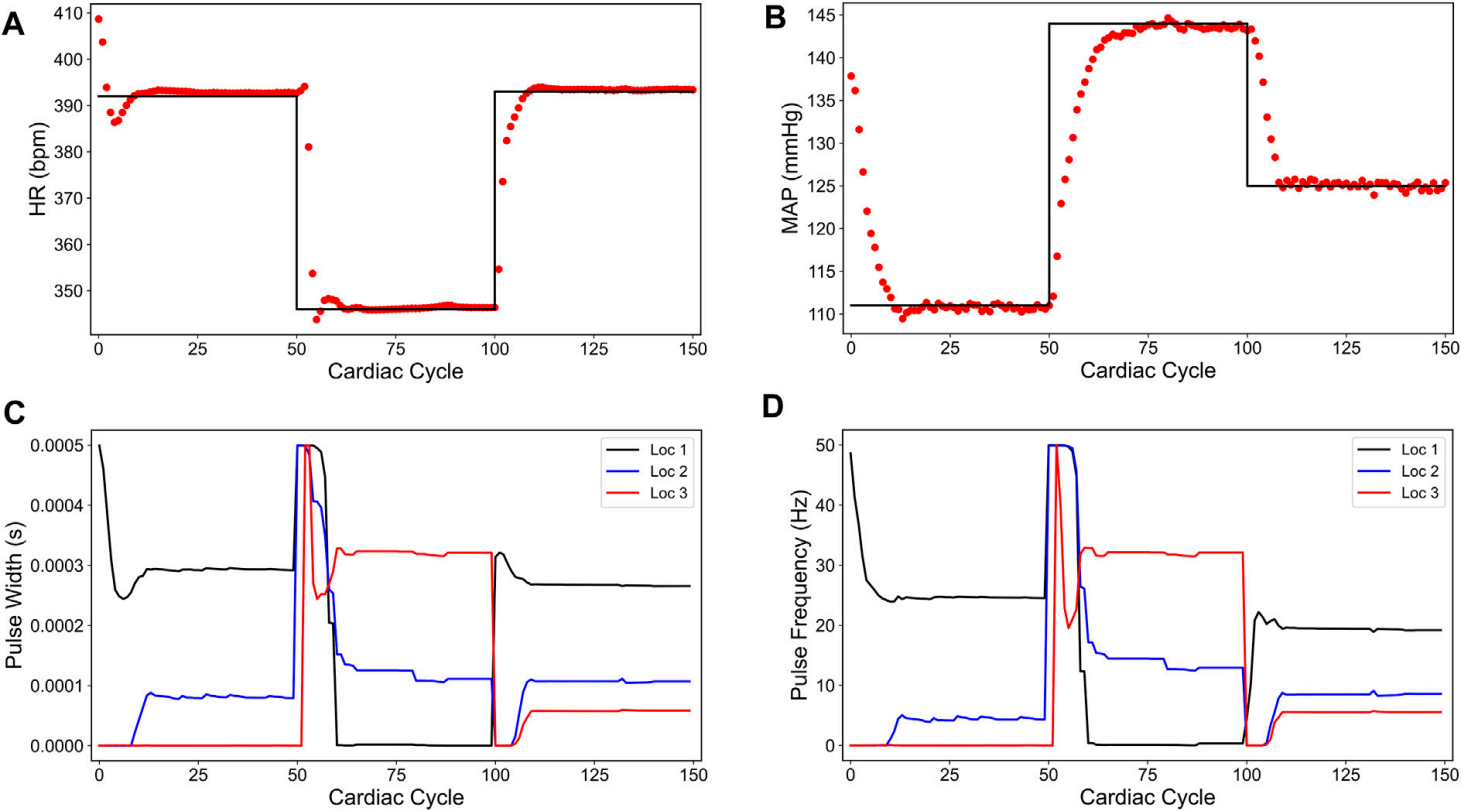
Minimum overshoot-based closed-loop control of the heart rate (HR) and the mean arterial blood pressure (MAP) of the *in silico* physiological model of the rat cardiac system. **(A)** and **(B)** show the closed-loop control response from the *in silico* physiological model (shown in red dots) and the target (or reference) for the controller (shown in black line) for HR and MAP, respectively. **(C)** and **(D)** show the pulse width and the stimulation frequency, respectively, designed by the controller at each VNS location.

Figures 5A,B show the controller performance in driving HR and MAP of the *in silico* physiological model to the desired set points. As shown here, adding an input difference term (the last term in the right hand side of the cost function (6)) minimized the previously seen fluctuations in MAP and leads to a smoother behavior in both HR and MAP compared to the sparsity-promoted and minimum energy-based strategies. Notably, this strategy resulted in no overshoot in the last set point for HR and a minimal overshoot for the other two target set points for HR.

The impact of the input difference term in the cost function (6) becomes more pronounced in the controller actions, as depicted in Figures 5C,D for VNS pulse width and frequency, respectively. We particularly found minimal to no oscillations in the controller actions as compared to the sparsity promoted and minimum energy-based strategies. Interestingly, the minimum overshoot based controller does not achieve the sparse solution at all target set points, evidenced by having all three locations active for the last set point.

In summary, we showed a data-driven model-based closed-loop control strategy to optimize the VNS parameters with multiple stimulation location sites, leading to smooth selection of VNS parameters in controlling cardiac physiology efficiently. This strategy may find applications in developing efficient closed-loop VNS therapy for cardiac diseases by avoiding overshoots/fluctuations in the controlled outputs.

### 2.3 Controlling Individual Patient Mismatch

So far, we have only considered scenarios where the developed LSTM model accurately predicted the heart rate (HR) and the mean arterial blood pressure (MAP) of the *in silico* physiological model of the rat cardiovascular system in response to VNS. We then used this LSTM model to design control strategies to optimize the VNS parameters to achieve a desired HR and MAP in the *in silico* physiological model. We demonstrated that the designed controller could efficiently drive HR and MAP to the desired set points as long as the LSTM predictions are reasonably accurate compared to the *in silico* physiological model (i.e., there is a minimal or no model mismatch between the LTSM and the *in silico* physiological model). In general, it may not always be feasible to obtain an accurate system model, mainly if experimental/clinical data are used to develop a system model. Should a reasonably good quantitative model be developed from the available experimental data, there may always be subject-to-subject variability in response to VNS which could potentially result in a significant model mismatch between the developed model and the physiological system. This model mismatch could lead to degraded performance of the designed controller in driving HR and MAP to the desired set points.

**FIGURE 6:**
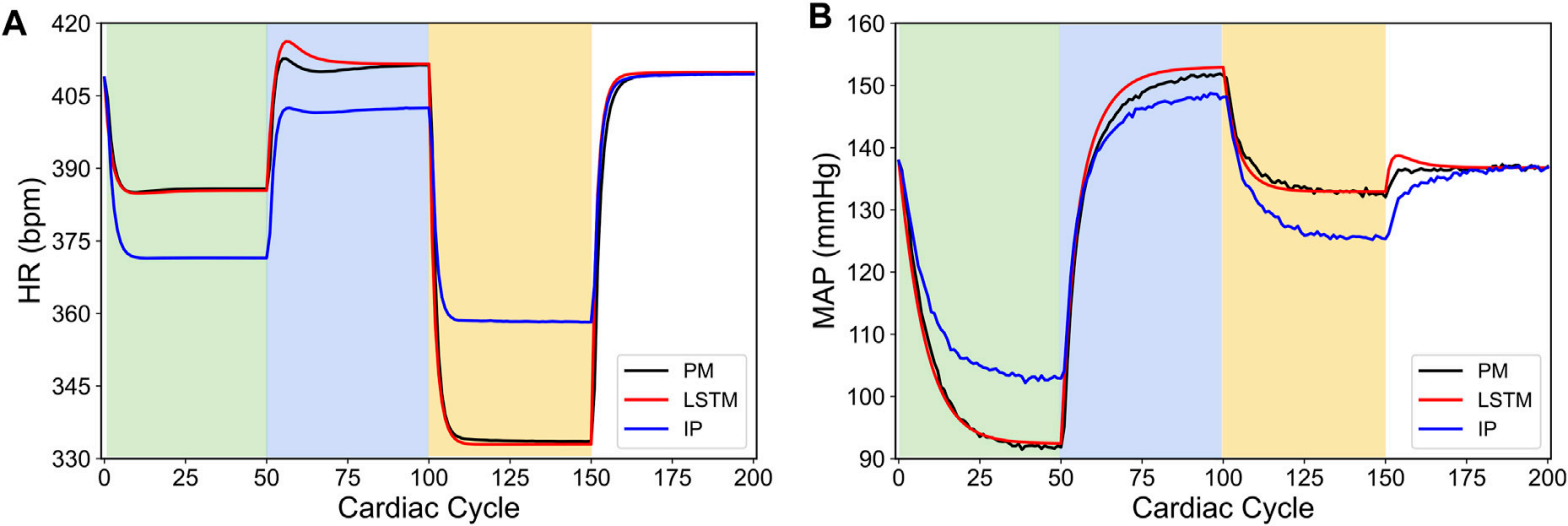
Comparison between the physiological outputs predicted from the modified *in silico* physiological model (IP, shown in blue), the *in silico* physiological model (PM, shown in black) and LSTM model (shown in red) in response to the vagus nerve stimulation (VNS) for 200 simulated cardiac cycles. **(A)** shows the heart rate (HR, bpm) and **(B)** shows the mean arterial blood pressure (MAP, mmHg). For cycles 1–50 (light green), location 1 was active with a pulse width of 0.36 ms and pulse frequency of 31 Hz. For cycles 51–100 (light blue), location 2 was active with a pulse width 0.19 ms and a pulse frequency 17 Hz. For cycles 101–150 (yellow), location 3 was active with a pulse width of 0.42 ms and a pulse frequency of 47 Hz. The last 50 cycles did not have any locations active.

To illustrate this point, we created a specific case where we modified the neuronal fiber recruitment concentrations at each VNS location in the *in silico* physiological model of the rat cardiovascular system (Yao and Kothare, 2020). Specifically, we changed the neuronal fiber concentrations at each location (*C*_*i*,*j*_) whose impact affects the output of Eq. 20, and ultimately leads to a different influence of VNS on the cardiac variables when compared to the baseline *in silico* physiological model. Figure 6 shows the predicted HR and MAP from the modified *in silico* physiological model in response to various VNS parameters and their comparison with the LSTM predicted values and the original *in silico* physiological model. As shown here, although the LSTM model did not predict the physiological HR and MAP quantitatively in response to VNS for the modified *in silico* physiological model, it indeed predicted the HR and MAP qualitatively. As noted in Figure 6 for the cardiac cycles between 150 and 200, the LSTM predicted both HR and MAP quantitatively with reasonable accuracy in the absence of VNS, which we expect since the modified *in silico* physiological model only differs from the original *in silico* physiological model (Yao and Kothare, 2020) in terms of how it responds to VNS.

**FIGURE 7:**
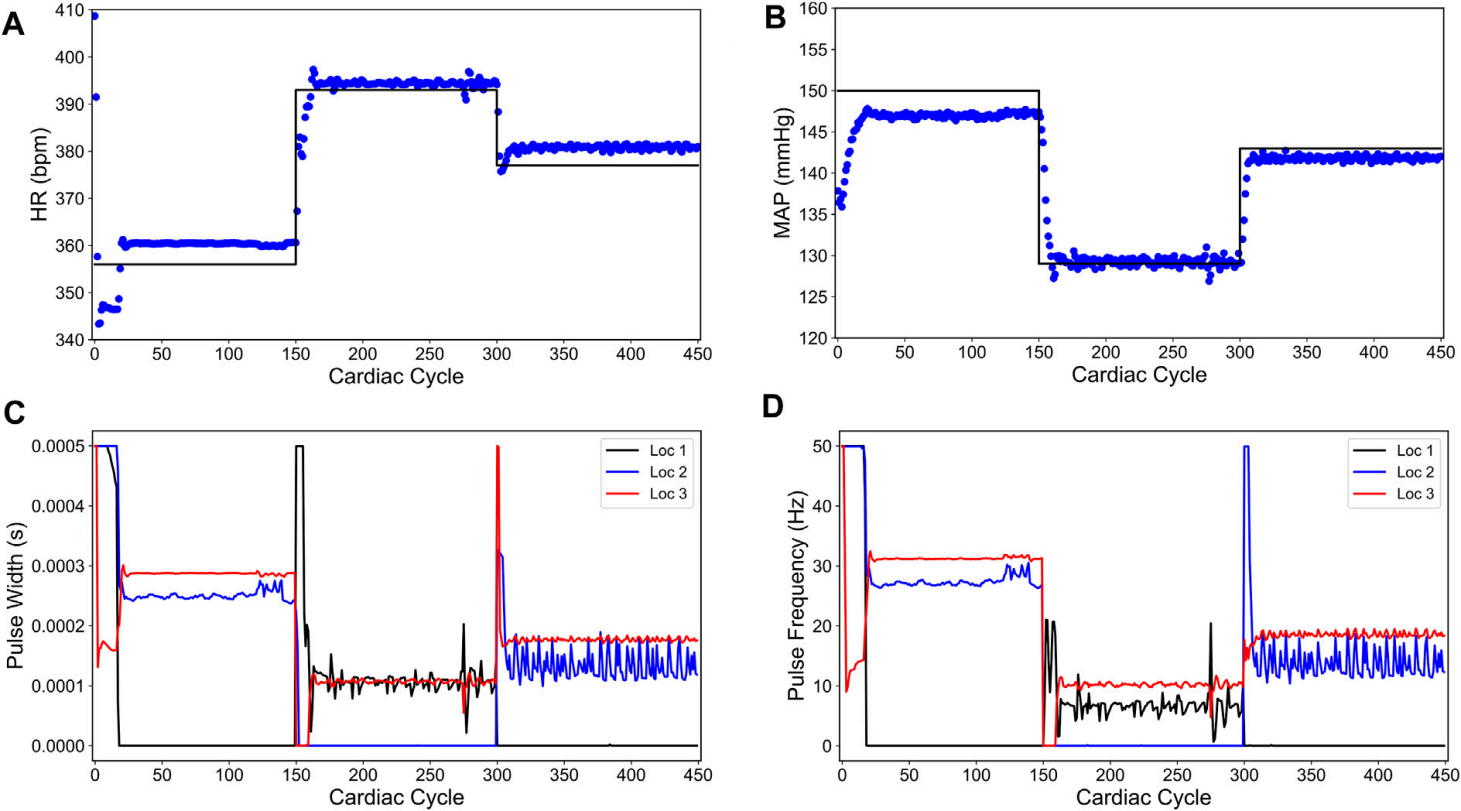
Sparsity-promoted closed-loop control of the heart rate (HR) and the mean arterial blood pressure (MAP) of the modified *in silico* physiological model of the rat cardiac system. **(A)** and **(B)** show the closed-loop control response from the modified *in silico* physiological model (shown in blue dots) and the target (or reference) for the controller (shown in black line) for HR and MAP, respectively. **(C)** and **(D)** show the pulse width and the stimulation frequency, respectively, designed by the controller at each VNS location.

We then used the same control formulation as described in the sparsity-promoted control design (see Section 2.2.1) but with the modified *in silico* physiological model in the closed-loop instead of the original *in silico* physiological model. Figure 7 shows the closed-loop performance of the designed controller. As noted in Figures 7A,B, the designed controller failed to drive HR and MAP to the desired set points. Specifically, the designed controller led to significant steady-state offset at most of the set-points. Interestingly, the sparsity promoted controller drove MAP to the desired set point for the cardiac cycles 150–300. This surprising result highlights one of the inherent advantages of model-based predictive control, wherein the feedback to the model provides inherent integral action to compensate for offsets.

To eliminate the steady-state offsets in the closed-loop performance, we adopted an approach of nonlinear offset-free control (Morari and Maeder, 2012) and reformulated our MPC optimization problem as follows:

$$\begin{array}{cccc} & \min & & J\left(k\right),\\ & \begin{array}{c} \vec{u}\left(k+j-1|k\right),\;j=1,2,\ldots ,N_{c}\\ {\vec{u}}_{s},{\hat{\vec{y}}}_{s} \end{array}\\ & s.t. & & {\vec{u}}_{min}\leq \vec{u}\left(k+j-1|k\right)\leq {\vec{u}}_{max},j\in \left[1,N_{c}\right],\\ & & & \hat{\vec{y}}\left(k+i|k\right)=f_{NN,aug}\left(\hat{\vec{y}}\left(k+i-1|k\right),\vec{d}\left(k\right),\vec{u}\left(k+i-1|k\right)\right),i\in \left[1,N_{p}\right],\\ & & & \hat{\vec{y}}\left(k|k\right)={\vec{y}}_{0}\left(k\right)\\ & & & \vec{r}\left(k\right)=f_{NN,aug}\left({\hat{\vec{y}}}_{s},\vec{d}\left(k\right),{\vec{u}}_{s}\right). \end{array}$$ (7)

Here, $\vec{u}\left(k+j|k\right)\in R^{6\times 1}$ is a vector of VNS parameters at the discrete time *k* + *j*, given that HR and MAP were measured from the modified *in silico* physiological model at time *k*. ${\vec{u}}_{\min}$ and ${\vec{u}}_{\max}$ are the minimum and maximum bound on the VNS parameters, respectively. $\hat{\vec{y}}\left(k+i|k\right)\in R^{2\times 1}$ is a vector of outputs (HR and MAP) predicted by the LSTM model at the discrete time *k* + *i*, given that HR and MAP were measured from the modified *in silico* physiological model (the physiological system to be controlled in our case) at time *k*. The function *f*_*NN*,*aug*_(⋅) represents the LSTM model augmented with a disturbance model (see Eq. 8b). *N*_*p*_ and *N*_*c*_ are the prediction and control horizon, respectively. ${\vec{y}}_{0}\left(k\right)\in R^{2\times 1}$ is the output (HR and MAP) measured from the *in silico* physiological model at time *k* in response to the optimal VNS parameters $\vec{u}\left(k-1|k-1\right)$ . $\vec{r}\left(k\right)\in R^{2\times 1}$ is a vector consisting of the reference or target HR and MAP. The vector $\vec{d}\left(k\right)\in R^{2\times 1}$ is a disturbance term which models the mismatch between the LSTM and the *in silico* physiological model at time *k* (see Eq. 8c). For a given $\vec{r}\left(k\right)$ and $\vec{d}\left(k\right)$ , ${\hat{\vec{y}}}_{s}$ and ${\vec{u}}_{s}$ are the steady-state solution of the disturbance-augmented LSTM model.

The optimization problem (7) is notably different from the previously formulated optimization problem (1) in two ways. First, this new optimization problem aims to minimize a scalar cost function J(k) with respect to two steady-state variables ${\vec{u}}_{s}\in R^{6\times 1}$ and ${\hat{\vec{y}}}_{s}\in R^{2\times 1}$ in addition to the VNS parameters $\vec{u}\left(k+j-1|k\right)\in R^{6\times 1}$ over the control horizon *N*_*c*_ used in the optimization problem (1). And second, we introduced an additional constraint in the optimization problem formulation which ensures that the current target $\left(\vec{r}\left(k\right)\right)$ is returned when the disturbance-augmented LSTM is evaluated at the steady-state optimized variables ${\vec{y}}_{s}$ and ${\vec{u}}_{s}$ for a given $\vec{d}\left(k\right)$ .

To account for the model mismatch between the output predictions from the LSTM model and the modified *in silico* physiological model (the physiological system to be controlled in our case) in the computation of optimal control actions, we introduced the following disturbance model:

$$f_{NN,aug}\left(\hat{\vec{y}}\left(k\right),\vec{d}\left(k\right),\vec{u}\left(k\right)\right)=f_{NN}\left(\hat{\vec{y}}\left(k\right),\vec{u}\left(k\right)\right)+\vec{d}\left(k\right)$$ (8a)

$$\vec{d}\left(k+1\right)=\vec{d}\left(k\right)+L_{d}\vec{\epsilon }\left(k\right)$$ (8b)

$$\vec{\epsilon }\left(k\right)=f_{NN,aug}\left(\hat{\vec{y}}\left(k-1\right),\vec{d}\left(k-1\right),\vec{u}\left(k-1\right)\right)-\vec{y}\left(k\right)$$ (8c)

Here, the augmented LSTM function $f_{NN,aug}\left(\hat{\vec{y}}\left(k\right),\vec{d}\left(k\right),\vec{u}\left(k\right)\right)$ described by Eq. 8a is the sum of the LSTM function $f_{NN}\left(\hat{\vec{y}}\left(k\right),\vec{u}\left(k\right)\right)$ and the disturbance variable ${\vec{d}}_{k}$ . The disturbance model (8c) updates the model mismatch between the measured outputs (HR and MAP) from the modified *in silico* physiological model and the predicted outputs from the LSTM model at the next time step by integrating a scaled error. Eq. 8c computes the model mismatch between the predicted outputs from the LSTM model and the measured outputs (HR and MAP) $\vec{y}\left(k\right)$ from the modified *in silico* physiological model at time *k*. $L_{d}\in R^{2\times 1}$ is a vector of constant gains.

At the beginning of the simulation, the disturbance variable $\vec{d}\left(0\right)$ was initialized to zero. To select the gain, *L*_*d*_ on the disturbance update model (8c), the authors in Morari and Maeder (2012) computed *L*_*d*_ based on the system model (well-behaved differential equation model in this case) linearized about the origin using the steady-state Kalman filter algorithm. In principle, the observer must be nominally asymptotically stable and satisfy: *L*_*d*_(*ϵ* = 0) = 0. In our case, such linearization was not possible for the LSTM model. Therefore, we selected the gain *L*_*d*_ based on a trial-and-error procedure. Particularly, we ran different trials of closed-loop simulations and examined the plot of the disturbance evolution over time. Typically, if a sufficiently inappropriate value was selected, the solver ceased to converge, often requiring a decrease in the *L*_*d*_ values. If the values were within the range required for the convergence, then the effect of increasing those values led to faster convergence to the steady-state solution. However, if the selected values were too large, then the error signal (Eq. 8c) was significantly emphasized, and the controller became unstable, often causing a failure in the convergence of the optimization solver. If the values of *L*_*d*_ were decreased, the controller took more iterations or a longer time to compensate for the offset. Based on this approach, we selected the following value of the gain *L*_*d*_:

$$L_{d}=\left[\begin{array}{cc} 0.06 & 0\\ 0 & 0.05 \end{array}\right]$$

We formulated the following cost function to be minimized within the optimization problem (7):

$$J\left(k\right)=\overset{N_{p}}{\underset{i=1}{\sum }}{\left({\hat{\vec{y}}}_{s}-\hat{\vec{y}}\left(k+i|k\right)\right)}^{T}Q\left({\hat{\vec{y}}}_{s}-\hat{\vec{y}}\left(k+i|k\right)\right)+\overset{N_{c}-1}{\underset{j=0}{\sum }}{\left({\vec{u}}_{s}-\vec{u}\left(k+j|k\right)\right)}^{T}R\left({\vec{u}}_{s}-\vec{u}\left(k+j|k\right)\right).$$ (9)

Here, the first summation term is the squared difference between the LSTM model predictions and the optimized variable ${\hat{\vec{y}}}_{s}$ . Note that this optimized variable represents the new target set point for the controller that accounts for the model mismatch. The second summation term is the squared difference between the control input and the optimized variable ${\vec{u}}_{s}$ . Again, note that this additional optimized variable is the actuation that together with ${\hat{\vec{y}}}_{s}$ satisfies the last constraint in the optimization problem (7) for a given $\vec{r}\left(k\right)$ and $\vec{d}\left(k\right)$ . Consistent with the previous cost functions, *Q* and *R* were set to an identity matrix of the appropriate dimensions. The constrained optimization problem (Eq. 7) together with the disturbance update (Eq. 8b), and the cost function (Eq. 9) enable the designed controller to achieve the steady-state offset free control of HR and MAP.

**FIGURE 8:**
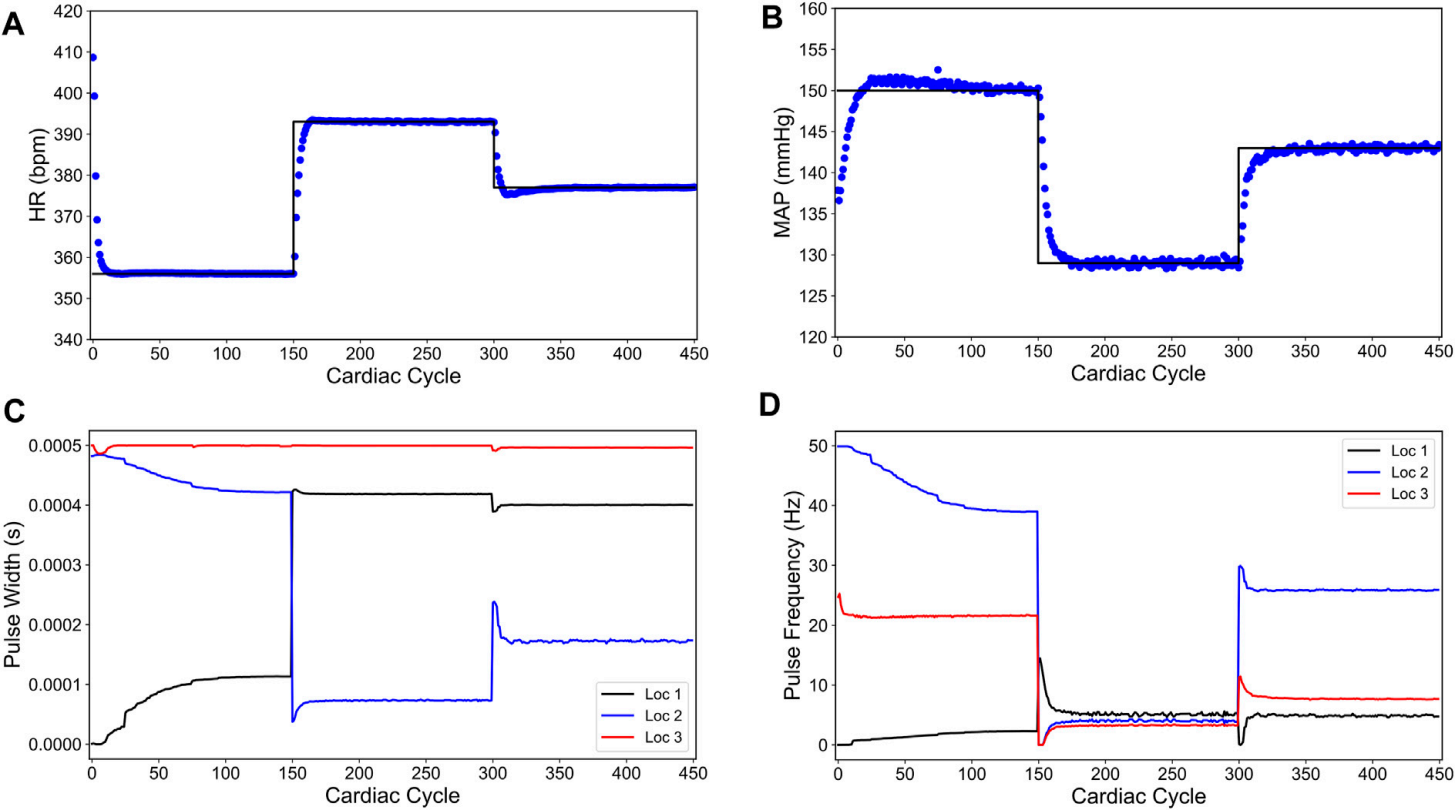
Offset-free closed-loop control of the heart rate (HR) and the mean arterial blood pressure (MAP) of the modified *in silico* physiological model of the rat cardiac system in the presence of intra-patient variability in the VNS response to HR and MAP. **(A)** and **(B)** show the closed-loop control response from the modified *in silico* physiological model (shown in blue dots) and the target (or reference) for the controller (shown in black line) for HR and MAP, respectively. **(C)** and **(D)** show the pulse width and the stimulation frequency, respectively, designed by the controller at each VNS location.

Figure 8 shows the closed-loop performance of our designed controller. For the first 150 cardiac cycles, the set point $\vec{r}\left(k\right)$ was set to 356 (bpm) and 150 (mmHg) for HR and MAP, respectively. For the next 150 cardiac cycles, the set point was set to 393 (bpm) and 129 (mmHg) for HR and MAP, respectively. Finally, for the final 150 cardiac cycles, the set point was set to 377 (bpm) and 143 (mmHg) for HR and MAP, respectively. The initial values were 409 (bpm) and 138 (mmHg) for HR and MAP, respectively. For the controller, we set *N*_*p*_ = 20 and *N*_*c*_ = 10 for this simulation. For each set point, we ran the simulation for 100 cardiac cycles longer (150 cardiac cycles as opposed to 50 cardiac cycles in previous closed-loop simulations) to ensure the convergence of the offset-free closed-loop control formulation.

As shown in Figure 8, the controller is able to achieve offset-free control within the first 50 cardiac cycles of each set point change. As the controller approaches each set point, there is an overshoot of each target for the first 20 cardiac cycles of the set point change. Notably, the optimized VNS parameters are generally smooth and constant near the steady-state.

Concisely summarized, we developed a closed-loop optimal control approach that accounts for the intra-patient variability of vagus nerve stimulation (VNS) response to the heart rate (HR) and the mean arterial blood pressure (MAP) in optimizing the VNS parameters for controlling HR and MAP. This specific closed-loop control formulation is appropriate in clinical/experimental settings where intra-patient variability is critical in designing VNS strategies, and the response of the implanted VNS device is sufficiently different from the responses used to train the LSTM model. A notable limitation of this approach is that it requires the controller’s task to be a set point tracking problem due to the offset-free constraint formulation.

### 2.4 Closed-Loop Control of Overactive Sympathetic System

**FIGURE 9:**
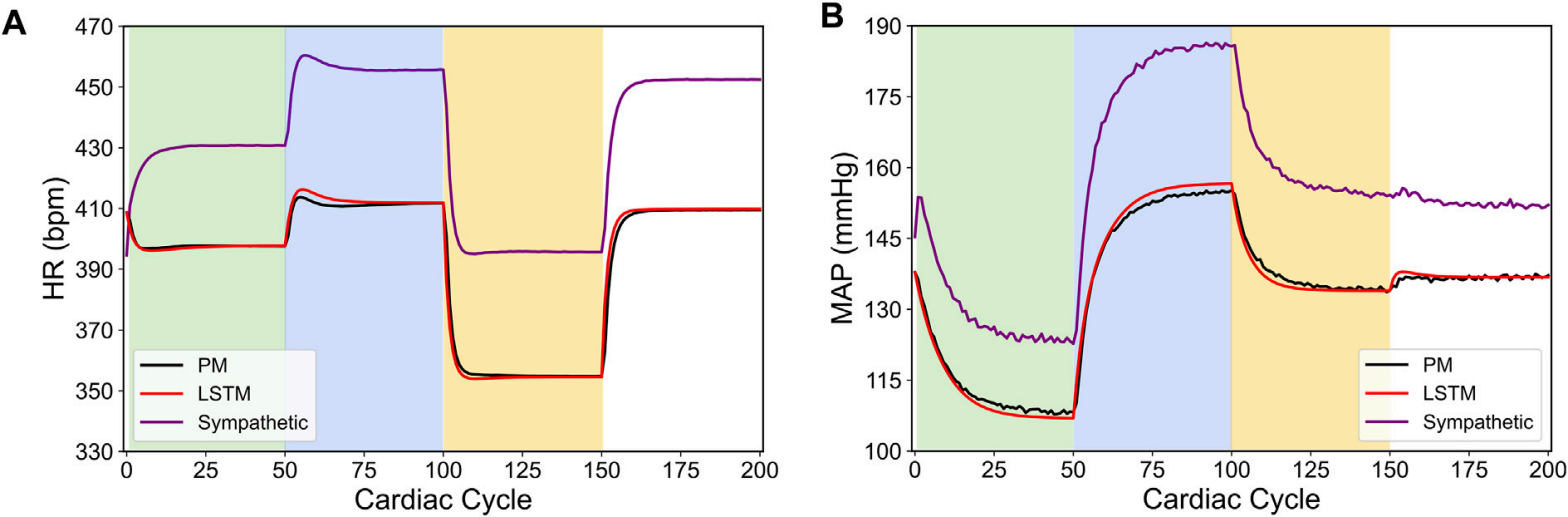
Comparison between the physiological outputs predicted from the modified *in silico* physiological model of an overactive sympathetic system (Sympathetic, shown in purple), the original *in silico* physiological model (PM, shown in black) and LSTM model (shown in red) in response to the vagus nerve stimulation (VNS) for 200 simulated cardiac cycles. **(A)** shows the heart rate (HR, bpm) and **(B)** shows the mean arterial blood pressure (MAP, mmHg). For cycles 1–50 (light green), location 1 was active with a pulse width of 0.36 ms and pulse frequency of 31 Hz. For cycles 51–100 (light blue), location 2 was active with a pulse width 0.19 ms and a pulse frequency 17 Hz. For cycles 101–150 (yellow), location 3 was active with a pulse width of 0.42 ms and a pulse frequency of 47 Hz. The last 50 cycles did not have any locations active.

To demonstrate whether the designed control strategy presented in the previous section could be used to regulate the heart rate (HR) and the mean arterial blood pressure (MAP) in a diseased case, we constructed an example of overactive sympathetic pathway case in the cardiac system. Specifically, we modified the parameters of the original *in silico* physiological model (Yao and Kothare, 2020) in a way such that the response to the sympathetic nervous system dominated over the parasympathetic response. These changes led to a higher resting heart rate, and a higher blood pressure, both of which are consistent with the behavior of some cardiovascular diseases (Malpas, 2010). We provide the specific parameter changes in Table 2. Particularly, this autonomic nervous system imbalance promotes heart failure, and vagal nerve stimulation has been suggested as a treatment therapy (Kishi, 2012). Similarly, the dominance of the sympathetic system corresponds to the increased blood pressure present in hypertension (Carthy, 2014). Additionally, hypertension has been shown to occur in parallel with other cardiovascular diseases (Palatini and Julius, 2009). Figure 9 shows the difference between this new *in silico* pathological behavior model and the original *in silico* physiological model. Notably, in the absence of VNS, the *in silico* pathological behavior model does not return to the same value as the original *in silico* physiological model, which is different from the intra-patient mismatch shown previously (compare with Figure 6).

**FIGURE 10:**
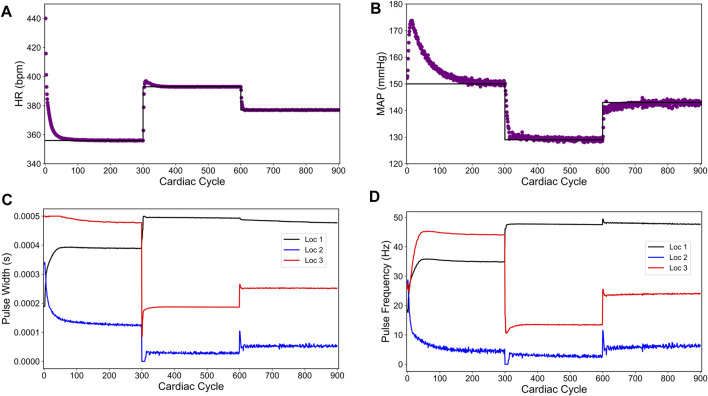
Offset-free closed-loop control of the heart rate (HR) and the mean arterial blood pressure (MAP) in an *in silico* overactive sympathetic system model of the rat cardiac system. **(A)** and **(B)** show the closed-loop control response from the modified *in silico* physiological model (shown in purple dots) and the target (or reference) for the controller (shown in black line) for HR and MAP, respectively. **(C)** and **(D)** show the pulse width and the stimulation frequency, respectively, designed by the controller at each VNS location.

To drive the HR and MAP to the desired set points, we applied the control formulation developed in the previous section (Eqs 7 and Eq. 8a, Eq. 8b, Eq. 8c, 9). Figure 10 shows the closed-loop controller performance for our designed controller. For this simulation, we set the initial heart rate to 452 bpm and the initial blood pressure to 152 (mmHg). For the first 300 cycles, the controller’s target was 356 (bpm) and 150 (mmHg) for HR and MAP, respectively. The following 300 cardiac cycles had the target changed to 393 (bpm) and 129 (mmHg), and the final 300 cardiac cycles the target was set to 377 (bpm) and 143 (mmHg). These are the same set points used for the intra-patient case (Figure 8). Regarding the controller, the controller parameters were set to the same previous values (*N*_*p*_ = 20 and *N*_*c*_ = 10). We set the disturbance gain vector *L*_*d*_ to

$$L_{d}=\left[\begin{array}{cc} 0.06 & 0\\ 0 & 0.018 \end{array}\right]$$

The length of simulation was considerably longer than the previous simulations (900 cardiac cycles compared to 450 cardiac cycles) because the disturbance variable, used for model mismatch compensation, took a longer time to reach the saturation due to a large model mismatch in this case. Nevertheless, the designed controller drove both HR and MAP to the desired set points with no steady-state offsets.

As noted in Figure 10B, the controller initially drives the MAP in the opposite direction of the target. This behavior is likely due to the significant model mismatch between the LSTM model and the modified *in silico* physiological model for the overactive sympathetic case. After about 50 cardiac cycles, the disturbance variable starts compensating for the mismatch and the MAP variable starts moving toward the target. After about 220 cardiac cycles, the disturbance variable has saturated leading to offset free control. As discussed previously, the MAP variable exhibits inherent fluctuations, and these fluctuations have grown larger with the new parameter selection. Even with the inherent model fluctuations, the MAP variable mean hits all of the set points and thus providing an offset-free control. Figures 10C,D show the optimal pulse width and stimulation frequency selected by the controller, respectively.

In summary, we modified the *in silico* physiological model of the rat cardiovascular system (the system to be controlled in our case) to exhibit increased responses in the sympathetic nervous system and decreased responses in the parasympathetic nervous system, and demonstrated that the offset-free controller formulation reached several set point tracking targets without a steady-state offset. This proof-of-concept suggests that a controller can be designed for cases where the diseased hemodynamics are considerably different from the hemodynamics used to train the LSTM or other neural network model to be used in designing a model-based optimal controller.

### 2.5 Impact of Prediction and Control Horizons on the Controller Performance and Computational Cost

In previous sections, we demonstrated various closed-loop optimal VNS designs for controlling the heart rate (HR) and the mean arterial blood pressure (MAP). In this section, we systematically evaluated the computational time required for simulating closed-loop control designs presented in the previous sections. Additionally, we compared the effect of the prediction horizon *N*_*p*_ and the control horizon *N*_*c*_ on the computational time and the closed-loop performance. All the comparisons were performed on an INTEL(R) CORE I7-9700 CPU 3.00 GHz with 16.0 GB of RAM Desktop.

Table 1 shows a summary of the computational time required to simulate 100 cardiac cycles (shown in terms of Average Time/Cycle) in closed-loop under specific control policy along with the closed-loop performance in terms of the mean absolute steady-state error between the desired setpoint and the closed-loop controlled output computed over the last 10 cardiac cycles for each setpoint. We estimated the computational time by taking the total time to run the closed-loop simulation for 100 cardiac cycles and subtracting the time it took the full *in silico* physiological model (the cardiac system to be controlled in our case) to simulate the number of cycles and dividing this difference by the total number of cycles in the simulation. For the sake of comparison, we set the parameter *λ*, *λ*_1_, and *λ*_2_ in the optimization problems to 0.001. As expected, when the control horizon *N*_*c*_ was increased, the computational time increased as well. A longer control horizon led to longer times for optimization to converge because the number of optimized variables increased by a six-fold rate as there were six additional parameters for the controller to optimize for every additional control horizon. Similarly, as the predictive horizon *N*_*p*_ increased, the time of optimization also increased as there were more predictions to be computed from the LSTM model. However, this additional computational time was not as significant compared to the increase in the control horizon *N*_*c*_. It should be noted that there were 8 additional variables to be optimized in the case of offset-free VNS design and overactive sympathetic case due to the different optimization problem formulation.

**TABLE 1:** Comparison of computational time and closed-loop performance of the designed closed-loop VNS strategies. Simulations were performed on an INTEL(R) CORE I7-9700 CPU 3.00 GHZ with 16.0 GB of RAM Desktop.

Nc (Cycles)	Np (Cycles)	Variables	Est. Time/Cycle (s)	SS MAE
Sparsity-promoted VNS design (Section 2.2.1)				
1	10	6	0.21	2.41
5	10	30	0.89	0.96
10	10	60	1.15	0.99
1	20	6	0.57	5.46
5	20	30	1.89	0.80
10	20	60	2.35	0.75
20	20	120	2.75	1.10
Minimum energy-based VNS design (Section 2.2.2)				
1	10	6	0.24	2.52
5	10	30	0.81	0.87
10	10	60	0.97	0.84
5	20	30	1.72	0.92
10	20	60	2.2	0.86
20	20	120	2.33	0.79
Minimum overshoot-based VNS design (Section 2.2.3)				
1	10	6	0.33	1.43
5	10	30	1.00	1.40
10	10	60	1.55	2.12
10	20	60	3.81	2.04
20	20	120	6.34	2.32
Offset free VNS design (Section 2.3)				
10	20	68	1.03	0.34
Overactive Sympathetic Case (Section 2.4)				
10	20	68	1.06	0.70

To compare the closed-loop performance over different prediction and control horizons across the implemented control strategies, we computed the steady-state mean absolute error (SS MAE) by taking the average of the mean absolute difference between the controlled output and the target setpoints for the last 10 cycles of each setpoint. It should be noted that SS MAE represents the combined absolute mean error of both the heart rate and the mean arterial blood pressure. We noted in Table 1 that the performance of the controller decreased to the point of showing a clear steady-state offset from the setpoints for the control horizon *N*_*c*_ = 1. It is expected that an increase in both *N*_*c*_ or *N*_*p*_ should exhibit less offset. This trend is not entirely shown by the results in Table 1 because the weights of the cost function (i.e., *λ*, *λ*_1_, *λ*_2_) were held constant. These results highlight how the selection of controller parameters can lead to different performances. Thus, the controllers with higher values of *N*_*c*_ or *N*_*p*_ likely need to be tuned to obtain comparable performance.

Based on the results shown in Table 1, we conclude that it is likely that selections with *N*_*c*_ ≥ 5 and *N*_*p*_ ≥ 20 would be difficult to implement in real-time due to large computational time. This leaves *N*_*c*_ ≤ 5, and *N*_*p*_ ≤ 10 as plausible choices for the real-time implementation of our closed-loop VNS designs. This analysis is based on the estimation that the controller would be continuously optimizing with roughly one second to optimize the result. In the case of *N*_*c*_ > 1, control actions can be implemented one at a time from the previous step optimization in case the next optimization has not converged in time.

## 3 Discussion

In this paper, we developed a model-based predictive closed-loop optimal control framework that utilizes a data-driven machine learning model of a system in optimizing vagus nerve stimulation (VNS) parameters to control the cardiovascular physiological outputs such as heart rate (HR) and mean arterial blood pressure (MAP). Using a synthetic dataset generated from a previously published *in silico* physiological model of the rat cardiovascular system (Yao and Kothare, 2020), we developed a long short-term memory (LSTM) based neural network model to predict HR and MAP of the *in silico* physiological model in response to VNS. The predictions of HR and MAP from our LSTM model showed quantitative consistency with the *in silico* physiological model in response to VNS with reasonable accuracy. We then used this model in designing various model-based optimal control strategies and demonstrated the efficacy of our closed-loop optimal control designs in controlling HR and MAP of the *in silico* physiological model of the rat cardiovascular system (the physiological system to be controlled in our case) in simulation. Finally, we showed in the simulation how our control designs could address intra-patient variability in closed-loop VNS designs and control HR and MAP in pathological conditions (e.g., overactive sympathetic cardiac system) where the model mismatch between the LSTM model and the system to be controlled is significant. Overall, our results highlight the advantage of using a closed-loop model predictive optimal control framework in optimizing VNS parameters to control multiple cardiac biomarkers.

Throughout our closed-loop designs and simulations, we chose appropriate design parameters such as the control and prediction horizons and various weighting matrices to obtain the best performances. Regarding the selection of these control design parameters, there are some guiding principles to select these parameters based on the observed closed-loop response of the designed controller. For example, in the case of the sparsity-promoted design presented in Section 2.2.1, a large value of *λ* instructs the controller to emphasize suppressing the inputs more than reaching the target value. A significant offset would evidence the occurrence of such a scenario in the controlled system’s outputs. Similarly, in the minimum energy-based design presented in Section 2.2.2, a large value of *λ* can lead to a similar situation where the controller is more focused on minimizing the energy than driving the outputs to the desired target.

As for this study, we have not found any significant difference in the closed-loop performance for different choices of prediction and control horizons, as shown in Section 2.5. However, we had noted that the computational time increased by approximately 3 times when we changed the prediction and control horizons from 10 and 5, respectively, to 20 and 20 (see Section 2.5). In general, the choice of control horizon *N*_*c*_ and prediction horizon *N*_*p*_ can have a significant impact on the closed-loop stability, performance, and computational time. Typically, a large prediction horizon can help in stabilizing the closed-loop system, while a large control horizon (less than or equal to the length of the prediction horizon) can provide less aggressive closed-loop performance. However, selecting large values for these parameters could also lead to large computational time to simulate the closed-loop response of the system, which may prevent the real-time implementation of the closed-loop control design in experiments.

Our optimal control problem formulation in this paper is in the form of a multi-objective optimization problem (Gunantara, 2018), where we have chosen these weights (*Q* and *R*) to be identity matrices of appropriate dimensions. The matrix *Q* allows to weigh specific output of interest more than other outputs to be controlled, and thus the optimization problem focuses more on minimizing this specific output than other outputs. Similarly, the matrix *R* allows to weigh specific input of interest more than other inputs to be optimized, and thus the optimization problem emphasizes minimizing this specific input compared to other inputs. Previously, it has been shown that artificial neural networks in model predictive control have improved the performance of biventricular assist devices for the treatment of heart failure (Ng et al., 2018). Similarly, our results have indicated that this approach allows for performance improvements.

The closed-loop control framework developed in this work provides flexibility in designing a control strategy for a specific situation. The optimization problem formulation can incorporate any relevant cost function and physiological constraints depending on the specific application. Additionally, as shown in Sections 2.3 and 2.4, the control problem formulation allows using the LSTM model trained on the data obtained from one subject to be applied in designing control strategy for a different subject, thus allowing a possibility to develop a model from the healthy subjects and use the model in designing control strategy for treating diseased cases.

While we showed the efficacy of the designed closed-loop VNS strategies in controlling the cardiac physiological biomarkers in an *in silico* physiological model of the rat cardiovascular system, the inclusion of the neural network permits the flexibility of developing a model of an *in vivo* physiological system as well. For example, consider an *in vivo* system where a single location VNS device with paramters of pulse amplitude, pulse width, and pulse frequency are used to control the heart rate. To provide the neural network with the data required to predict this relationship, open-loop trials involving the selection of a VNS pulse (i.e., the selection of pulse amplitude, pulse width, and pulse frequency) are applied according to a predefined duty cycle while recording the heart rate. With this dataset, a neural network can be trained using the change in the heart rate while the VNS device is on to learn the effect of VNS parameters on the cardiovascular system. While it does not necessarily matter in which order the VNS stimulation parameters are chosen, there is reason to gradually increase the parameters for ease of subject comfort (Ardell et al., 2017). Additionally, collecting open-loop data over several days to assess temporal variation (analgous to different initial conditions in the *in silico* physiological model) may improve the accuracy of the neural network. Similarly, the neural network’s predictive performance may improve through data collection during different states of the subject (i.e., stress, level of physical activity, etc.), provided this can be done so safely. These additional considerations seek to provide the neural network with the variety of data required to tune its predictions based on the different operating regimes of the cardiovascular system, which will likely improve the performance of the accompanying controller.

Should these controller designs be deployed in the experimental/clinical setup, there is a potential that the controller could switch its objective function to meet the demands of the physiological system. In a therapeutic context, stimulating all locations at all times could lead to a loss in the efficacy of specific treatment. Conversely, there may be times when power consumption holds a higher priority than enforcing a sparse solution. For the therapeutic application, the closed-loop control formulation in Section 2.2.3 could drive the physiological system to reach the desired targets with more smooth transitions, which may be more gentle for the patient or subject of the therapy. These different contexts are why we have investigated multiple controller designs in this paper. Importantly, all the control designs have been shown to reach the target set point with a nominal to no steady-state offset. Their similar performances suggest a potential of employing them in the experimental system while accounting for multiple external factors (battery level, physiological feedback, stimulation time, etc.) to provide the context for selecting the specific controller design used at a particular time.

Due to limitations of the *in silico* physiological model, there are additional details concerning implementation that we could not investigate here. For instance, heart rate is not necessarily a directly measurable biomarker and is derived from the electrocardiograph (ECG) measurement, leading to the question of abstracting a sufficient correlation directly from data containing the ECG and the applied VNS parameters. The presence of noise in an *in vivo* system can be addressed through designing an observer to estimate the true values of the biomarkers of interest, although this has not been demonstrated here. As a consequence of the *in silico* physiological model used for training the LSTM, we have omitted the pulse amplitude as an optimized VNS parameter. We anticipate that the inclusion of this parameter would not cause any fundamental problem to our approach. Additionally, most of the published studies on optimizing VNS for cardiac system did not consider the influence of disease pathology on controller performance (Zhang et al., 2002; Tosato et al., 2006; Ugalde et al., 2015; Greenwald et al., 2016; Romero-Ugalde et al., 2017; Yao and Kothare, 2020). While we have considered this by modifying the physiological dynamics (see Sections 2.3 and 2.4), it is worth noting that the offset-free controller formulation is limited to overcoming the steady-state offsets in the closed-loop performance. Thus, should the controller’s objective change to a tracking problem (as opposed to a constant set point), then the controller’s performance would not necessarily achieve nominal offset in the presence of model mismatch. While this limitation may appear exceptionally prohibitive, depending on the application of the VNS device, such a limitation may not restrict all applications. Additionally, assessing the controller performance when training a neural network with open-loop data from multiple subjects as opposed to a single subject could lead to insight regarding a generalized way to model the *in vivo* system. These topics are under active investigation and comprise future work.

## 4 Methods

### 4.1 *In Silico* Rat Cardiac Model

We used a previously published *in silico* physiological model of the rat cardiac system that includes the effect of two VNS parameters (pulse width in ms and pulse frequency in Hz) on the two physiological variables (heart rate (HR) in bpm and mean arterial blood pressure (MAP) in mmHg) (Yao and Kothare, 2020). Here, we provide a brief description of this *in silico* physiological model and refer the reader to this study for a full model description. The model was composed of three different parts: the cardiovascular system, the baroreflex system, and the vagus nerve stimulation (VNS) device. Regarding the VNS device, there were three different locations to apply VNS, following the experimental setup of Plachta et al. (2014), and thus there were six total VNS parameters (three locations with two parameters each). To determine the effects of VNS on the physiological outputs, each location contained a concentration of vagal, baroreceptive, and sympathetic neuronal fibers. The application of VNS led to different fiber recruitment levels depending on the parameters chosen. From the fiber recruitment, the effect on the physiological variables were determined through the interactions between the simulated central nervous system, and the cardiac system.

#### 4.1.1 Cardiovascular Model

Design of the cardiovascular system were based on the previously published models (Djabella et al., 2005; Ferreira et al., 2005). Parameters for the cardiovascular system came from (Ferreira et al., 2005) and were adjusted by the body volume ratio of rats to humans, resulting in values similar to those measured in experimental rats (Pacher et al., 2004). The pressure volume relationship was described as

$$P_{i}=E_{i}\left(V_{i}-V_{i,d}\right),$$ (10)

where the instantaneous blood pressure of compartment *i* is denoted by *P*_*i*_, the total volume is denoted by *V*_*i*_, unstressed volume is denoted by *V*_*i*,*d*_, and the elastance is denoted by *E*_*i*_. The elastance, *E*(*t*), was described, by following the formulation of (Stergiopulos et al., 1996), as:

$$E\left(t\right)=E_{max}\left(a\;\frac{{\left(\frac{t_{n}}{\alpha_{1}T}\right)}^{n_{1}}}{1+{\left(\frac{t_{n}}{\alpha_{1}T}\right)}^{n_{1}}}\;\frac{1}{{\left(\frac{t_{n}}{\alpha_{2}T}\right)}^{n_{2}}}\right)+E_{min},$$ (11)

where *t*_*n*_ denotes the periodic time, *T* denotes the cardiac period, *E*_max_ denotes the end-systolic elastance, *E*_min_ denotes the end-diastolic elastance, and *a*, *α*_1_, *α*_2_, *n*_1_, *n*_2_ are all dimensionless constants. The flow, *Q*, between chambers of the cardiac system was modeled as follows:

$$Q=\frac{P_{in}-P_{\mathrm{out}}}{C_{i}R_{\mathrm{sys}}}.$$ (12)

Here, *P*_*in*_ and *P*_*out*_ represent the pressure difference causing blood flow, *C*_*i*_ represents a compliance constant, and *R*_*sys*_ represents the cardiac system’s resistance to blood flow. Following a mass balance, the change in volume was given by

$$\frac{dV}{dt}=Q_{in}-Q_{\mathrm{out}},$$ (13)

where *Q*_*in*_ represents the flow into the compliance chamber, and *Q*_*out*_ represents the flow out of the compliance chamber. Finally, the inertial flow out of the left ventricle was described as

$$\Delta P=L\frac{dQ}{dt},$$ (14)

where *L* denotes the inertance, and Δ*P* denotes the pressure difference due to the inertial blood flow.

#### 4.1.2 Baroreflex Model

The baroreflex system model was derived from (Lau and Figueroa, 2015), and was composed of several parts: the central nervous system, the baroreceptor model, and a modulation of efferent responses. Following the activation of the sympathetic drive, the modulation of efferent responses was described by the left ventricle systolic elastance (*E*_max_) and the cardiac resistance to blood flow (*R*_*sys*_) exhibiting a positive response, and conversely the heart period (*T*) exhibiting a negative response. A previously developed input-output relationship of the interactions between the central nervous system and baroreceptive fibers was used to determine the effect of the baroreflex system on cardiovascular functioning (Ursino, 1998). The parameters for the baroreceptive model were taken from (Mahdi et al., 2013), and the parameters of the baroreflex system model were taken from (Ursino, 1998). To keep generality, each location was assumed to be concentrated with 100% of a specific neuronal fiber type (i.e., location 1 was assumed to be concentrated with only baroreceptive fibers), which led to a qualitative match with the experimental data presented in (Plachta et al., 2014). The sympathetic efferent pathway was described using the following equation:

$$\frac{d\theta_{es}}{dt}=-\frac{1}{\tau_{\theta_{es}}}\left(\theta_{es}-\theta_{0}\right)+G_{\theta_{es}}\;\ln\left(\max\left\{f_{es}\left(t-D_{\theta_{es}}\right)-f_{es,min},1\right\}\right).$$ (15)

Here, *θ*_*es*_ denotes each of the efferent path variables: the heart period *T*, the systolic elastance of the left ventricle *E*_max_, and the inertial cardiac flow resistance, *R*_*sys*_. The baseline value in the absence of external input is given by *θ*_0_, $\tau_{\theta_{es}}$ denotes the time constant, *f*_*es*_(*t*) represents the firing rate of the sympathetic efferents, $G_{\theta_{es}}$ denotes the gain, $D_{\theta_{es}}$ denotes the delay of the effector, and *f*_*es*, min_ denotes the minimum firing rate of the sympathetic efferents. Following the activation of vagal fibers, the first-order dynamics were used to capture the corresponding change in the heart period as follows:

$$\frac{dT_{ev}}{dt}=-\frac{1}{\tau_{T_{ev}}}\left(T_{ev}-T_{0}\right)+G_{T_{ev}}\;f_{ev}\left(t-D_{T_{ev}}\right).$$ (16)

Here, *T*_*ev*_ denotes the change in the heart period due to activation of vagal fibers, *T*_0_ denotes the resting heart period, $\tau_{T_{ev}}$ is the time constant, $G_{T_{ev}}$ is the gain of the heart period, and $D_{T_{ev}}$ is the delay of the vagal pathway. The effects on the heart period (*T*) from sympathetic activation were assumed to be independent of the effects from the vagal activation, leading to the following calculation for the heart period from the total effects of stimulation:

$$T=T_{ev}+T_{es}-T_{0}.$$ (17)

Here, *T*_*ev*_ denotes the effect of vagal (parasympathetic) efferents on the heart period, *T*_*es*_ denotes the effect of sympathetic efferents on the heart period, and *T*_0_ is the resting heart period.

#### 4.1.3 VNS Device Model

The stimulation device translates VNS parameter selection into neural firing rate changes, with an assumption that the device increases the firing rates of the baroreceptive fibers, efferent sympathetic fibers, and vagal fibers. The fiber recruitment due to pulse width was given by

$$F\left(P_{w}^{i}\right)=\frac{P_{w}^{i}/k_{w}}{\sqrt{1+{\left(P_{w}^{i}/k_{w}\right)}^{2}}},$$ (18)

where *i* = 1, 2, … , *n* is the location index, *k*_*w*_ denotes a dimensionless scaling parameter, $P_{w}^{i}$ denotes the pulse width, and $F\left(P_{w}^{i}\right)$ denotes the fiber recruitment at each location. The change in firing rates due to pulse frequency was given by

$$\Delta R\left(P_{f}^{i}\right)=\frac{P_{f}^{i}/k_{f}}{\sqrt{1+{\left(P_{f}^{i}/k_{f}\right)}^{2}}},$$ (19)

where *i* = 1, 2 … , *n* denotes the location index, *k*_*f*_ denotes a dimensionless scaling parameter, $P_{f}^{i}$ denotes the pulse frequency, and $\Delta R\left(P_{f}^{i}\right)$ denotes the change in firing rates of each fiber. Since the change in fiber firing rates leads to the change in physiological variables, this aggregate effect of fiber recruitment regarding the selection of the pulse width and the pulse frequency on the firing rates of neuronal fiber type *j* was described by

$$\Delta f_{j}=\frac{G_{j}}{n}\overset{n}{\underset{i=1}{\sum }}\delta_{i}C_{i,j}F\left(P_{w}^{i}\right)\Delta R\left(P_{f}^{i}\right).$$ (20)

Here, *i* = 1, 2, …, *n* is the location index, *j* = 1, 2, 3 indicates the fiber type index, *δ*_*i*_ indicates an on/off of the *i*th location, *C*_*i*,*j*_ represents the concentration of fiber type *j* at location *i*. The gain of each fiber’s excitability is represented by *G*_*j*_, and *f*_*j*_ denotes the final change in the firing rate of the fibers.

Thus, Eqs 10–20, together describe the complete physiological dynamics of the *in silico* rat cardiac system with the influence of VNS.

### 4.2 Intra-Patient Variation Cardiac Model

In Plachta et al. (2014), the authors showed that there is a significant variation across rats in response to the vagus nerve stimulation (VNS) parameter selection. Particularly in their study, they showed that the qualitative response to the specific stimulation parameters on the heart rate was consistent across the animals, but there were significant statistical variations in the quantitative responses (i.e., a 20% decrease in heart rate was observed in one rat, while a 10% decrease in the heart rate was observed in another rat for the same stimulation parameter selection). To demonstrate how our closed-loop VNS design can account for this subject-to-subject variability in response to VNS, we constructed an example by modifying the *in silico* physiological model of the rat cardiac system. Specifically, we modified the concentration of fiber recruitment at each VNS location (see *C* in Eq. 20) from an identity matrix to:

$$C=\left[\begin{array}{ccc} 1.1 & 0.1 & 0.2\\ 0.1 & 0.8 & 0.0\\ 0.2 & 0.2 & 0.6 \end{array}\right]$$

Here, the row in the matrix *C* represents the specific location, and the column represents the fiber type recruited at that location. For example, row 1 in the matrix *C* indicates that the VNS location 1 activates 110% of barorecpetive fiber, 10% of sympathetic fibers, and 20% of vagal fibers compared to the baseline responses. Note that these values were set to an identity matrix in the *in silico* physiological model, which says that each location has a 100% concentration of an individual fiber type (baroreceptive, sympathetic, and vagal). Since this modification in the *in silico* physiological model only affects the actuation side of the model (i.e., the VNS effect), the model mismatch shown in Figure 6 is due to the stimulation parameters exhibiting differential effects on the physiological variables.

### 4.3 Overactive Sympathetic Cardiac Model

Often, throughout the progression of specific cardiovascular disease pathology, the sympathetic system becomes overactive at the resting state (Malpas, 2010). This hyperactivity is typically observed by a higher resting heart rate, a higher resting blood pressure, or both, depending on the specific disease considered. Since VNS is a therapy that targets disease pathology, we questioned if a closed-loop VNS control design could perform through a potential mismatch in the hypothetical case of an overactive sympathetic system. To investigate this question, we constructed a hypothetical example of an overactive sympathetic case where we modified the parameters of the *in silico* physiological model of rat cardiac system to increase the influence of the sympathetic system and decrease the influence from the parasympathetic system, consistent with disease pathology that exhibits a reduced parasympathetic tone. We summarize the specific changes we made in the *in silico* physiological model parameters, described in Section 4.1, in Table 2.

**TABLE 2:** Description of the modified parameters in the *in silico* physiological model of rat cardiovascular system Yao and Kothare (2020) to construct an overactive sympathetic case.

Parameters	Description	Initial value	Diseased value	Equations
*R*1	systemic resistance	0.01	0.007	15
*R*2	mitral valve resistance	0.0001	0.0002	12
*R*3	aortic valve resistance	0.008	0.006	12
*C*2	veneous compliance	20	25	12
*C*3	systemic compliance	1.8	1.4	12
*E*_min_	end-diastolic elastance	0.02	0.01	11
*E*_max_	end-systolic elastance	1.2	1.1	11
*T*_0_	baseline HR	60/450	60/480	17
*G*_*R*_	gain of systemic resistance	0.06	0.07	15
*G*_*Ts*_	gain of heart period from sympathetic fibers	−0.01	−0.015	15
$G_{T_{ev}}$	gain of heart period from vagal fibers	0.015	0.011	16
*G*_2_	gain of sympathetic fibers	30	33	20
*G*_3_	gain of vagal fibers	30	27	20

The behavior of the *in silico* physiological model has changed considerably with the new parameter selection, as demonstrated by Figure 9. As expected, the physiological cardiac model with overactive sympathetic system exhibited a higher heart rate and higher blood pressure. In addition to the changes in the model parameters shown in Table 2, we also changed the concentration of fiber recruitment at each VNS location (see *C* in Eq. 20) from an identity matrix to:

$$C=\left[\begin{array}{ccc} 1.3 & 0.1 & 0.1\\ 0.1 & 1.4 & 0.1\\ 0.1 & 0.1 & 1.3 \end{array}\right]$$

By changing the actuation matrix of the model and the model parameters, we considered both the intra-patient model mismatch and the disease model mismatch. Should a controller be deployed in the clinical setting, the controller would likely be required to compensate for both sources of mismatch simultaneously.

### 4.4 LSTM Model Development

We recently developed a purely data-driven long short-term memory (LSTM) based neural network modeling approach to map the VNS parameters on the cardiac physiology (Branen et al., 2021). LSTM is the state-of-art in data-driven modeling of dynamical systems and sequence based tasks (Hochreiter and Schmidhuber, 1997) demonstrated by its use for forecasting traffic patterns (Zhao et al., 2017), natural language processing (Radford et al., 2019), handwriting recognition (Graves et al., 2008), and speech recognition (Sak et al., 2014). The LSTM uses a combination of a hidden state, a cell state, and the incoming data to predict the sequential evolution of the system. The inclusion of the cell state allows for learning of long-scale temporal dynamics, and is managed by a gating process where incoming data with the previous hidden state is used to forget a portion of the previous information from the cell state. Additionally, an input gate allows the relevant information from the incoming data and previous hidden state to be stored in the cell state. The output from a LSTM is based on the updated cell state, previous hidden state, and the incoming data.

In this work, we used a LSTM based modeling approach to predict the effect of VNS parameters on the dynamics of the heart rate (HR) and the mean arterial blood pressure (MAP) (Branen et al., 2021). We generated a synthetic input-output dataset by simulating the *in silico* physiological rat cardiac model (Eqs 10–20) for 15,198 individual trials using VNS parameters from a randomly sampled uniform distribution with a range between 0 and 0.5 ms for the pulse width and 0 and 50 Hz for the pulse frequency. Each location was also turned on/off by sampling from a uniform random distribution between 0 and 1. The specific location is turned off if the selected value is 0 and turned on if the selected value is 1. An individual trial consists of 100 consecutive cardiac cycles with constant VNS parameters. These open loop trials were then used to train the neural network using a 40%–20%–40% split for the training, validation, and testing sets respectively. We note that this synthetically generated dataset represents a similar task to real data collection methods, and could be extended to an *in vivo* experimental system where different experimental trials are used to generate a sweep of the VNS parameters. In the case of data collection from the *in vivo* system, random sampling of VNS parameters and locations is not required, and can be replaced with a systematic investigation of the parameter space, such that the full range of parameters have been swept. The LSTM was trained for 250 epochs using the adaptive moment estimation (Adam) optimizer (Kingma and Ba, 2014) with a mean squared error loss function. Prior to feeding the data to the LSTM for training, the open-loop dataset was normalized using the following equation:

$$\hat{x}=\frac{x-\mu }{x_{max}-x_{min}}$$ (21)

where *μ* is the mean of the training set, *x*_min_ is the minimum of the training set, and *x*_max_ is the maximum value of the training set for each variable. Statistics from the training set are used to avoid providing any information about the validation or test datasets to the trained network, as this is a standard practice in the machine learning community. As a note, since the LSTM was trained in the normalized space, all control applications required incoming data for predictions to be normalized, and the controller actions to be un-normalized before applying to the *in silico* physiological model during closed-loop operation.

**FIGURE 11:**
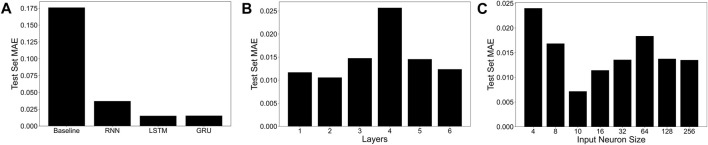
Performance comparison of different trained neural network architectures based on the mean absolute error for the same test set. **(A)** shows a comparison of baseline performance to a traditional (or conventional) RNN, gated recurrent unit (GRU) and LSTM. **(B)** Shows the a comparison of the effect of the number of LSTM layers on the model’s predictive performance. **(C)** Shows a comparison of the effect of the number of inputs on the model’s predictive performance.

To compare different trained architectures, the test set of data was used in such a way that the trained network was required to recursively predict 99 consecutive cardiac cycles (1 trial) for both HR and MAP. A baseline model that predicted no change in the initial value for all 99 cycles was used to provide context to the normalized mean absolute error (MAE) and emphasize a trained network’s ability to learn the dynamics. These results are summarized in Figure 11, with different recurrent networks (GRU is a gated recurrent unit) shown in Figure 11A, the influence of number of LSTM layers on predictive performance is shown in Figure 11B, and the influence of LSTM input neuron size shown in Figure 11C.

To conclude the study of several different neural network architectures, the best trained model resulted in a LSTM with a single layer, hidden dimensionality of 10, and a hyperbolic tangent (tanh) activation function. Hyperbolic tangent is a common nonlinear function used in machine learning, and is centered about 0 (*x* = 0, tanh(*x*) = 0) and demonstrates saturating nonlinearity with very large inputs (*x* > 5 or *x* < − 5) saturating at output values of 1 and −1, respectively. The output from this LSTM was fed to a dense layer with dimensionality of two, and tanh activation function. The performance of this LSTM is highlighted by a normalized mean absolute error of 0.0072 on the test set. A prediction from the LSTM for time varying VNS inputs is shown in Figure 1. The LSTM model reasonably predicts the output from the full *in silico* physiological model, and is capable of mapping VNS parameters to the effect on the cardiac variables of heart rate and mean arterial blood pressure.

### 4.5 Simulation Environment and Relevant Software Packages

Throughout this work, we used MATLAB (version R2019b) to simulate the *in silico* physiological model of rat cardiac system. The LSTM model was developed in Python (version 3.7) using TensorFlow2. The controller was synthesized in Python (version 3.7) and the formulated optimization problems were solved using the sequential least squares programming (SLSQP) algorithm in Scipy (version 1.6). The closed-loop simulations were performed through the interaction of Python with MATLAB. For the healthy cases, the optimization solver’s tolerance was set to 10*e* − 5 and the maximum number of iterations was set to 50. While, for the intra-patient and diseased cases, the solver’s tolerance was set to 10*e* − 4 and the maximum number of iterations was set to 500 (although the algorithm converged in less than 50 iterations in our simulations).

## Data Availability

The code used in this study can be found at Github https://github.com/Bradrew/Data-Driven-Control-of-VNS-Cardiac.git.

## References

[B1] Agnew W. F. McCreery D. B. Yuen T. G. H. Bullara L. A. (1989). Histologic and Physiologic Evaluation of Electrically Stimulated Peripheral Nerve: Considerations for the Selection of Parameters. Ann. Biomed. Eng. 17, 39–60. 10.1007/bf02364272 2537589

[B2] Ardell J. L. Nier H. Hammer M. Southerland E. M. Ardell C. L. Beaumont E. (2017). Defining the Neural Fulcrum for Chronic Vagus Nerve Stimulation: Implications for Integrated Cardiac Control. J. Physiol. 595, 6887–6903. 10.1113/jp274678 28862330 PMC5685838

[B3] Asad Z. U. Stavrakis S. (2019). Vagus Nerve Stimulation for the Treatment of Heart Failure. Bioelectron. Med. 2, 43–54. 10.2217/bem-2019-0012

[B4] Benjamin E. J. Virani S. S. Callaway C. W. Chamberlain A. M. Chang A. R. Cheng S. (2018). Heart Disease and Stroke Statistics-2018 Update: A Report from the American Heart Association. Circulation 137, e67–e492. 10.1161/CIR.0000000000000558 29386200

[B5] Branen A. Yao Y. Kothare M. V. Mahmoudi B. Kumar G. (2021). Mapping Vagus Nerve Stimulation Parameters to Cardiac Physiology Using Long Short-Term Memory Network. Annu. Int. Conf. IEEE Eng. Med. Biol. Soc. 2021, 5477–5480. 10.1109/EMBC46164.2021.9630667 34892365

[B6] Capilupi M. J. Kerath S. M. Becker L. B. (2020). Vagus Nerve Stimulation and the Cardiovascular System. Cold Spring Harb. Perspect. Med. 10, a034173. 10.1101/cshperspect.a034173 31109966 PMC6996447

[B7] Carthy E. R. (2014). Autonomic Dysfunction in Essential Hypertension: a Systematic Review. Ann. Med. Surg. 3, 2–7. 10.1016/j.amsu.2013.11.002 PMC4268473 25568776

[B8] Djabella K. Médigue C. Sorine M. (2005). “A Differential Model of the Baroreflex Control of the Cardiovascular System during a Tilt Test,” in Proceedings of the 44th IEEE Conference on Decision and Control (Seville, Spain: IEEE), 903–908.

[B9] Ferreira A. Chen S. Simaan M. A. Boston J. R. Antaki J. F. (2005). “A Nonlinear State-Space Model of a Combined Cardiovascular System and a Rotary Pump,” in Proceedings of the 44th IEEE Conference on Decision and Control (Seville, Spain: IEEE), 897–902.

[B10] Gold M. R. Van Veldhuisen D. J. Hauptman P. J. Borggrefe M. Kubo S. H. Lieberman R. A. (2016). Vagus Nerve Stimulation for the Treatment of Heart Failure. J. Am. Coll. Cardiol. 68, 149–158. 10.1016/j.jacc.2016.03.525 27058909

[B11] Goodfellow I. Bengio Y. Courville A. (2016). Machine Learning Basics. Deep Learn. 1, 98–164.

[B12] Graves A. Liwicki M. Fernández S. Bertolami R. Bunke H. Schmidhuber J. (2008). A Novel Connectionist System for Unconstrained Handwriting Recognition. IEEE Trans. Pattern Anal. Mach. Intell. 31, 855–868. 10.1109/TPAMI.2008.137 19299860

[B13] Greenwald E. So E. Wang Q. Mollazadeh M. Maier C. Etienne-Cummings R. (2016). A Bidirectional Neural Interface Ic with Chopper Stabilized Bioadc Array and Charge Balanced Stimulator. IEEE Trans. Biomed. Circuits Syst. 10, 990–1002. 10.1109/tbcas.2016.2614845 27845676 PMC5258841

[B14] Gunantara N. (2018). A Review of Multi-Objective Optimization: Methods and its Applications. Cogent Eng. 5, 1502242. 10.1080/23311916.2018.1502242

[B15] Heidenreich P. A. Albert N. M. Allen L. A. Bluemke D. A. Butler J. Fonarow G. C. (2013). Forecasting the Impact of Heart Failure in the United States. Circ. Heart Fail. 6, 606–619. 10.1161/hhf.0b013e318291329a 23616602 PMC3908895

[B16] Hochreiter S. Schmidhuber J. (1997). Long Short-Term Memory. Neural Comput. 9, 1735–1780. 10.1162/neco.1997.9.8.1735 9377276

[B17] Howland R. H. (2014). Vagus Nerve Stimulation. Curr. Behav. Neurosci. Rep. 1, 64–73. 10.1007/s40473-014-0010-5 24834378 PMC4017164

[B50] Kwakernaak H. Sivan R. Tyreus B. N. D. (1974). Linear Optimal Control Systems.

[B18] Kingma D. P. Ba J. (2014). Adam: A Method for Stochastic Optimization. arXiv Prepr. arXiv:1412.6980.

[B19] Kishi T. (2012). Heart Failure as an Autonomic Nervous System Dysfunction. J. Cardiol. 59, 117–122. 10.1016/j.jjcc.2011.12.006 22341431

[B20] Kong W. Dong Z. Y. Jia Y. Hill D. J. Xu Y. Zhang Y. (2017). Short-term Residential Load Forecasting Based on Lstm Recurrent Neural Network. IEEE Trans. Smart Grid 10, 841–851.

[B21] Lau K. D. Figueroa C. A. (2015). Simulation of Short-Term Pressure Regulation during the Tilt Test in a Coupled 3D-0D Closed-Loop Model of the Circulation. Biomech. Model. Mechanobiol. 14, 915–929. 10.1007/s10237-014-0645-x 25567754 PMC4490186

[B22] Mahdi A. Sturdy J. Ottesen J. T. Olufsen M. S. (2013). Modeling the Afferent Dynamics of the Baroreflex Control System. PLoS Comput. Biol. 9, e1003384. 10.1371/journal.pcbi.1003384 24348231 PMC3861044

[B23] Malpas S. C. (2010). Sympathetic Nervous System Overactivity and its Role in the Development of Cardiovascular Disease. Physiol. Rev. 90, 513–557. 10.1152/physrev.00007.2009 20393193

[B24] Mangoni M. E. Traboulsie A. Leoni A.-L. Couette B. Marger L. Le Quang K. (2006). Bradycardia and Slowing of the Atrioventricular Conduction in Mice Lacking Ca V 3.1/α 1G T-type Calcium Channels. Circulation Res. 98, 1422–1430. 10.1161/01.res.0000225862.14314.49 16690884

[B25] Melchior F. M. Srinivasan R. S. Charles J. B. (1992). Mathematical Modeling of Human Cardiovascular System for Simulation of Orthostatic Response. Am. J. Physiology-Heart Circulatory Physiology 262, H1920–H1933. 10.1152/ajpheart.1992.262.6.h1920 1621848

[B26] Morari M. Maeder U. (2012). Nonlinear Offset-free Model Predictive Control. Automatica 48, 2059–2067. 10.1016/j.automatica.2012.06.038

[B27] Ng B. C. Salamonsen R. F. Gregory S. D. Stevens M. C. Wu Y. Mansouri M. (2018). Application of Multiobjective Neural Predictive Control to Biventricular Assistance Using Dual Rotary Blood Pumps. Biomed. Signal Process. Control 39, 81–93. 10.1016/j.bspc.2017.07.028

[B28] Ovbiagele B. Goldstein L. B. Higashida R. T. Howard V. J. Johnston S. C. Khavjou O. A. (2013). Forecasting the Future of Stroke in the United States. Stroke 44, 2361–2375. 10.1161/str.0b013e31829734f2 23697546

[B29] Pacher P. Mabley J. G. Liaudet L. Evgenov O. V. Marton A. Haskó G. (2004). Left Ventricular Pressure-Volume Relationship in a Rat Model of Advanced Aging-Associated Heart Failure. Am. J. Physiology-Heart Circulatory Physiology 287, H2132–H2137. 10.1152/ajpheart.00405.2004 PMC2756475 15231502

[B30] Palatini P. Julius S. (2009). The Role of Cardiac Autonomic Function in Hypertension and Cardiovascular Disease. Curr. Sci. Inc 11, 199–205. 10.1007/s11906-009-0035-4 19442329

[B31] Plachta D. T. T. Gierthmuehlen M. Cota O. Espinosa N. Boeser F. Herrera T. C. (2014). Blood Pressure Control with Selective Vagal Nerve Stimulation and Minimal Side Effects. J. Neural Eng. 11, 036011. 10.1088/1741-2560/11/3/036011 24809832

[B32] Plaster B. Kumar G. (2019). Data-driven Predictive Modeling of Neuronal Dynamics Using Long Short-Term Memory. Algorithms 12, 203. 10.3390/a12100203

[B33] Premchand R. K. Sharma K. Mittal S. Monteiro R. Dixit S. Libbus I. (2014). Autonomic Regulation Therapy via Left or Right Cervical Vagus Nerve Stimulation in Patients with Chronic Heart Failure: Results of the Anthem-Hf Trial. J. cardiac Fail. 20, 808–816. 10.1016/j.cardfail.2014.08.009 25187002

[B34] Radford A. Wu J. Child R. Luan D. Amodei D. Sutskever I. (2019). Language Models Are Unsupervised Multitask Learners. OpenAI blog 1, 9.

[B35] Romero-Ugalde H. M. Le Rolle V. Bonnet J.-L. Henry C. Bel A. Mabo P. (2017). A Novel Controller Based on State-Transition Models for Closed-Loop Vagus Nerve Stimulation: Application to Heart Rate Regulation. PloS one 12, e0186068. 10.1371/journal.pone.0186068 29077707 PMC5659642

[B36] Sahoo B. B. Jha R. Singh A. Kumar D. (2019). Long Short-Term Memory (Lstm) Recurrent Neural Network for Low-Flow Hydrological Time Series Forecasting. Acta Geophys. 67, 1471–1481. 10.1007/s11600-019-00330-1

[B37] Sak H. Senior A. W. Beaufays F. (2014). Long Short-Term Memory Recurrent Neural Network Architectures for Large Scale Acoustic Modeling.

[B38] Savarese G. Lund L. H. (2017). Global Public Health Burden of Heart Failure. Card. Fail. Rev. 03, 7. 10.15420/cfr.2016:25:2 PMC5494150 28785469

[B39] Stergiopulos N. Meister J. J. Westerhof N. (1996). Determinants of Stroke Volume and Systolic and Diastolic Aortic Pressure. Am. J. Physiology-Heart Circulatory Physiology 270, H2050–H2059. 10.1152/ajpheart.1996.270.6.h2050 8764256

[B40] Suga H. Sagawa K. Shoukas A. A. (1973). Load Independence of the Instantaneous Pressure-Volume Ratio of the Canine Left Ventricle and Effects of Epinephrine and Heart Rate on the Ratio. Circulation Res. 32, 314–322. 10.1161/01.res.32.3.314 4691336

[B41] Tosato M. Yoshida K. Toft E. Nekrasas V. Struijk J. J. (2006). Closed-loop Control of the Heart Rate by Electrical Stimulation of the Vagus Nerve. Med. Bio Eng. Comput. 44, 161–169. 10.1007/s11517-006-0037-1 16937157

[B42] Trespalacios F. Grossmann I. E. (2014). Review of Mixed-Integer Nonlinear and Generalized Disjunctive Programming Methods. Chem. Ing. Tech. 86, 991–1012. 10.1002/cite.201400037

[B43] Ugalde H. M. Ojeda D. Le Rolle V. Andreu D. Guiraud D. Bonnet J. L. (2015). Model-based Design and Experimental Validation of Control Modules for Neuromodulation Devices. IEEE Trans. Biomed. Eng. 63, 1551–1558. 10.1109/TBME.2015.2498878 26571507

[B44] Ursino M. (1998). Interaction between Carotid Baroregulation and the Pulsating Heart: a Mathematical Model. Am. J. Physiology-Heart Circulatory Physiology 275, H1733–H1747. 10.1152/ajpheart.1998.275.5.h1733 9815081

[B45] Vidaurre D. Bielza C. Larrañaga P. (2013). A Survey ofL1Regression. Int. Stat. Rev. 81, 361–387. 10.1111/insr.12023

[B46] Yao Y. Kothare M. V. (2020). “Model Predictive Control of Selective Vagal Nerve Stimulation for Regulating Cardiovascular System,” in 2020 American Control Conference (ACC) (Denver, CO, USA: IEEE), 563–568. 10.23919/acc45564.2020.9147771

[B47] Zannad F. De Ferrari G. M. Tuinenburg A. E. Wright D. Brugada J. Butter C. (2015). Chronic Vagal Stimulation for the Treatment of Low Ejection Fraction Heart Failure: Results of the Neural Cardiac Therapy for Heart Failure (Nectar-hf) Randomized Controlled Trial. Eur. Heart J. 36, 425–433. 10.1093/eurheartj/ehu345 25176942 PMC4328197

[B48] Zhang Y. Mowrey K. A. Zhuang S. Wallick D. W. Popović Z. B. Mazgalev T. N. (2002). Optimal Ventricular Rate Slowing during Atrial Fibrillation by Feedback Av Nodal-Selective Vagal Stimulation. Am. J. Physiology-Heart Circulatory Physiology 282, H1102–H1110. 10.1152/ajpheart.00738.2001 11834509

[B49] Zhao Z. Chen W. Wu X. Chen P. C. Y. Liu J. (2017). LSTM Network: a Deep Learning Approach for Short‐term Traffic Forecast. IET Intell. Transp. Syst. 11, 68–75. 10.1049/iet-its.2016.0208

